# The photometry and kinematics studies of NGC 2509 derived from Gaia DR3

**DOI:** 10.1038/s41598-025-00383-x

**Published:** 2025-05-21

**Authors:** Nasser M. Ahmed, A. L. Tadross

**Affiliations:** National Research Institute of Astronomy and Geophysics (NRIAG), Cairo, Helwan 11421 Egypt

**Keywords:** Star cluster, Gaia DR3, 2Mass, CMD, Parallax, Proper motion, Distance, Membership, Mathematics and computing, Optics and photonics, Astronomy and planetary science, Astronomy and astrophysics

## Abstract

This research uses the third edition of the Gaia Data Release (DR3) to re-investigate the open star cluster NGC 2509. We employed the pyUPMASK Python package and HDBSCAN algorithms to identify the cluster member stars. *The current analysis introduces a new method that connects the membership probability of stars in the cluster (using the pyUPMASK tool) with the number of stars predicted by the King model at different distances from the center of the cluster. This approach divides the cluster’s area into concentric rings, or shells, and calculates the membership probability for each shell based on its specific star count, rather than using one average probability for the entire cluster.* We calculated all astrophysical parameters of NGC 2509-including center, cluster radius, radial density distribution, color-magnitude diagram, distance, age, and reddening-using the photometric and astrometric data of Gaia DR3. The cluster’s relaxation time, total mass, luminosity, and mass functions are computed. The components of the proper motions ($\mu _{\alpha }\text {cos}\delta$, $\mu _{\delta }$), and the parallax ($\varpi$) are found to be $-2.710 \pm 0.142$, $0.802 \pm 0.146$mas/yr and $0.368 \pm 0.043$mas, respectively. According to the King model and pyUPMASK membership, we obtained $497 \pm 39$stars with a total mass of $491.2\pm 59.5$ $M_{\odot }$. Using the PARSEC stellar isochrones fit, the mean cluster age and its relaxation time are $1.72\pm 12.3$Gyr and $93.5 \pm 24.5$Myr, respectively. The cluster distance modulus and reddening are estimated to be $11.68\pm 0.12$, and $0.13 \pm 0.04$mag, resulting in a distance of $2168.5\pm 261.5$pc. The mass function MF for this cluster has been constructed using a piecewise powerlaw with two power laws, $\alpha _1$ and $\alpha _2$, rather than the single power law as suggested by Salpeter (1955). The $\alpha _1$ and $\alpha _2$ are found to be $-2.74 \pm 0.14$and $2.29 \pm 0.13$, respectively. Moreover, the $\alpha _2$ is closest to the Salpeter value. Also, we identified 20 member stars as red clump that have G magnitudes between 12.6 and 13.1 mag and slightly higher temperatures than typical giants. We found that 11 members are flagged as variable stars in Gaia DR3 archive.  In addition, there are 88 stars with a radial velocity of around 57.6 ± 7.8 km. Then, we have used the galpy Python package to calculate the cluster’s kinematics and orbital parameters.

## Introduction

Deciphering the formation history of the Milky Way’s disc requires an understanding of open star clusters (OCs), which are gravitationally bound groups of stars with comparable ages and chemical abundances. OCs are essential objects for advancing our knowledge about stellar evolution, kinematics, structure, and astrophysical properties of the Milky Way Galaxy^1–4^, including the spiral arms^5–7^, star formation processes^8–10^,  chemical composition, age, and metallicity estimations^11–17^. The Milky Way disk is populated by OCs that reveal a wide range of ages, from less than 100 Myr to approximately 8 Gyr^3,17–19^. The main astrophysical parameters of OCs, i.e., distance modulus, color excess (extinction), age, and metallicity can be determined from their color-magnitude diagrams (CMDs); by comparing observed data with isochrones of stellar models.

NGC 2509 is located in the northern Milky Way at 2000.0 equatorial coordinates of $\alpha =08^{h} \ 00^{m} \ 48^{s}, \delta =-19^{\circ } \ 03^{\prime } \ 06^{\prime \prime },$ and galactic coordinates of $\ell = 237.84^{\circ },\ b= +05.84^{\circ }$. It is located in the direction of the Puppies constellation, on the Orion arm of the Galaxy^20^. A historical review of NGC 2509 before 2005 has been given by Tadross (^21^and references therein), where he studied the most main photometrical properties based on the 2MASS database. Carraro and Costa^22^studied the main photometric parameters of NGC 2509 with another four clusters. Recently^23^, studied the atmospheric parameters of members of the cluster under study with another 15 such cluster as unstudied objects. Presently, Almeida et al.^24^revisited the mass of the investigated cluster with a large sample of such cluster using the Gaia database. Also^25^, studied the structural and fundamental astrophysical parameters of NGC 2509. However, the fundamental characteristics of NGC 2509 that collected from the literature can be summarized in Table 1.

**Table 1:** The available parameters of NGC 2509 were estimated in the previous studies.

Parameter	^21^	^22^	^3^	^24^	^25^	^26^	^27^	^19^	this work
Log age (yr)	9.2	9.08	9.18	9.15	9.17	9.25	9.18	–	9.23
Dist. (pc)	2000	2900	2495	2800	2518	2208	–	–	$2168.5\pm 261.5$
E(B-V) (mag)	–	–	–	–	–	0.1	–	–	–
$E(G_{BP}-G_{RP})$ (mag)	–	–	–	–	0.1	–	–	–	$0.13 \pm 0.04$
m-M (mag)	11.5	12.5	–	–	12.19	11.72	–	–	$11.68\pm 0.12$
Members	660	–	222	–	–	–	212	447	$497 \pm 39$
$M_{total} \ (M_{\odot })$	800	–	–	1454	–	–	–	–	$491.2\pm 59.5$
$\varpi$ (mas)	–	–	0.363	–	0.37	–	0.404	0.373	$0.368 \pm 0.043$
$\mu _{\alpha }\text {cos}\delta$ (mas/yr)	–	–	−2.708	–	−2.718	–	−2.718	−2.72	$-2.710 \pm 0.142$
$\mu _{\delta }$ (mas/yr)	–	–	0.764	–	0.803	–	0.798	0.8	$0.802 \pm 0.146$
Radius (arcmin)	8.0	2.5	–	–	–	–	–	–	$19.25\pm 3.25$
$R_{gc}$ (kpc)	9.7	10.3	9.9	–	–	–	–	–	9.69

The aim of this study is to provide a comprehensive photometric and astrometric analysis of the open cluster NGC 2509 using the Gaia DR3 database. This analysis enables us to derive key astrophysical parameters, e.g., mass, age, metallicity, and radius, as well as astrometric parameters, e.g., parallax, proper motion’s components, and the cluster’s dynamical evolution.

The paper is organized as follows: Section "Gaia DR3 data" outlines the criteria used to extract the initial data sample from Gaia DR3. The cluster’s structure, along with its radial density profile, is described in Section "Cluster density profile". In Section "Membership determination", We introduce our method for determining membership. The astrometric analysis of proper motions is presented in section "Proper motions, cluster kinematics and dynamics", leading to an investigation of the cluster’s kinematics and dynamics. The photometric properties of the cluster members are discussed in Sections "Photometric analysis of the cluster". Finally, the main conclusions are summarized in Section "Summary and conclusions".

## Gaia DR3 data

**Fig. 1:**
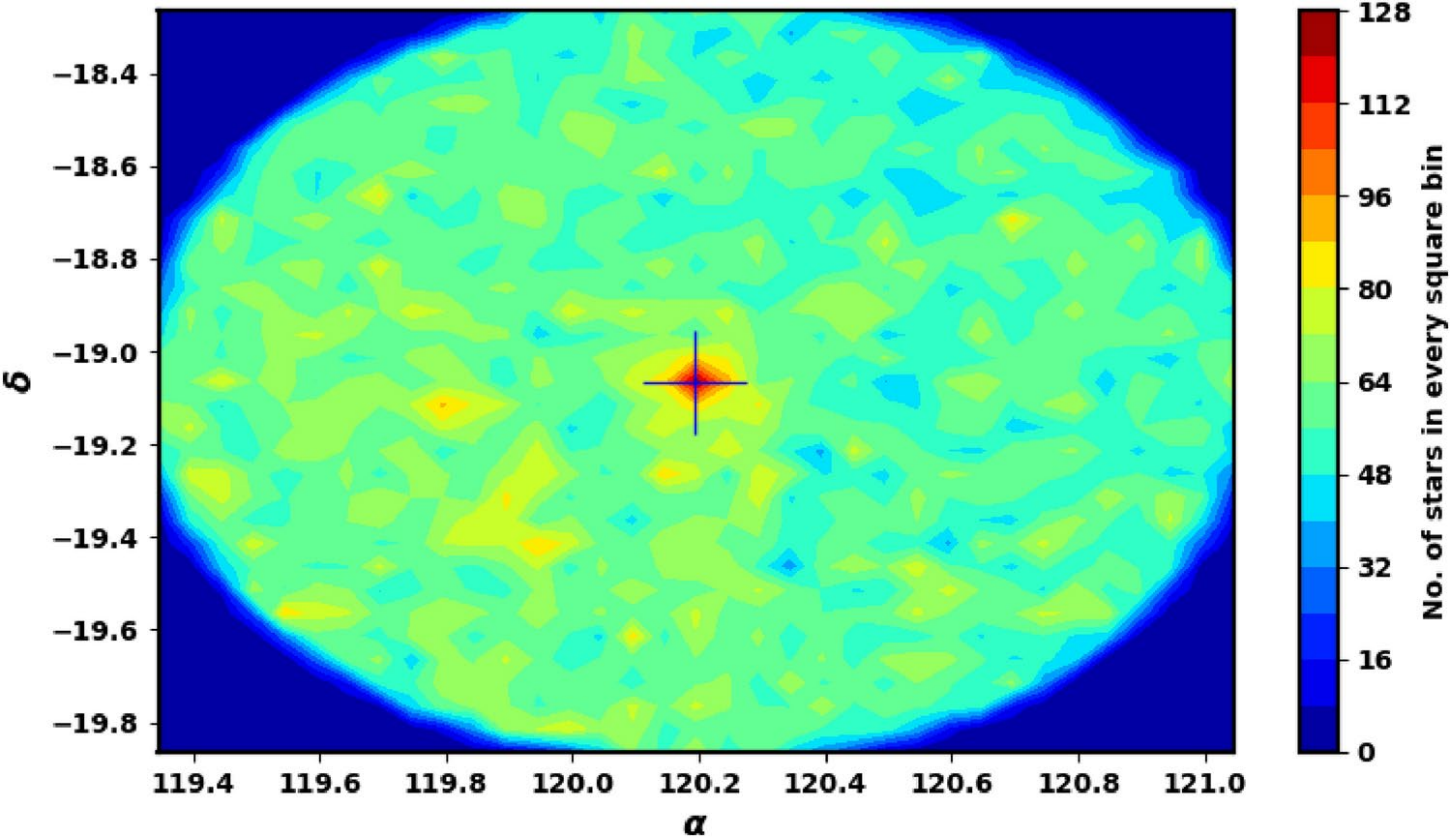
The number surface density of NGC 2509 using the data of Gaia DR3.

**Fig. 2:**
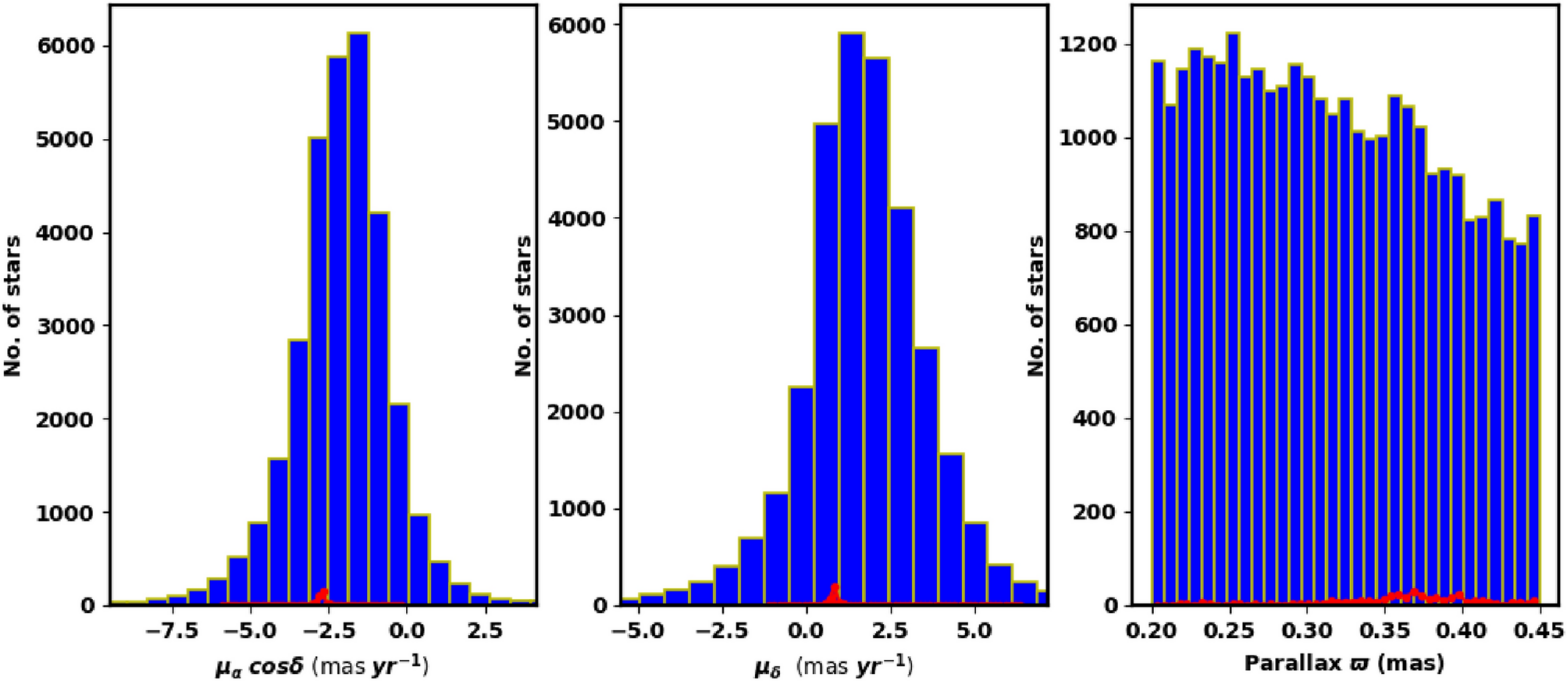
The proper motion in right ascension, declination ($\mu _{\alpha }\cos \delta$, $\mu _{\delta }$) and parallax ($\varpi$) in the field of NGC 2509.

We obtained the data for NGC 2509 from the Gaia DR3 catalog^27^. This dataset contains sky positions ($\alpha$, $\delta$), proper motions ($\mu _{\alpha }\cos \delta$, $\mu _{\delta }$), and parallaxes ($\varpi$), with a limiting magnitude of G = 21 mag. Gaia DR3 provides astrophysical parameters for a wide range of celestial objects derived from parallaxes, broad-band photometry, and mean radial velocity spectra. The parallax errors in Gaia DR3 range from 0.02 to 0.07 milli-arc-seconds (mas) for sources with G $\le$ 17 mag, increase to 0.5 mas at G = 20 mag, and reach 1.3 mas for sources at G = 21 mag. The range of proper motion errors is between 0.02 and $0.07\,\text {mas yr}^{-1}$ for G $\le$ 17 mag, reaching $0.5\,\text {mas yr}^{-1}$ for G = 20 mag, and up to $1.4\,\text {mas yr}^{-1}$ for G = 21 mag. The catalog includes G magnitudes for around 1.806 billion sources, $G_{BP}$ magnitudes for approximately 1.542 billion sources, and $G_{RP}$ magnitudes for around 1.555 billion sources. Fig. 1 shows the surface number density of NGC 2509 from Gaia DR3, while Fig. 2 presents the histograms for proper motions ($\mu _{\alpha }\cos \delta$, $\mu _{\delta }$) and parallax ($\varpi$).

**Fig. 3:**
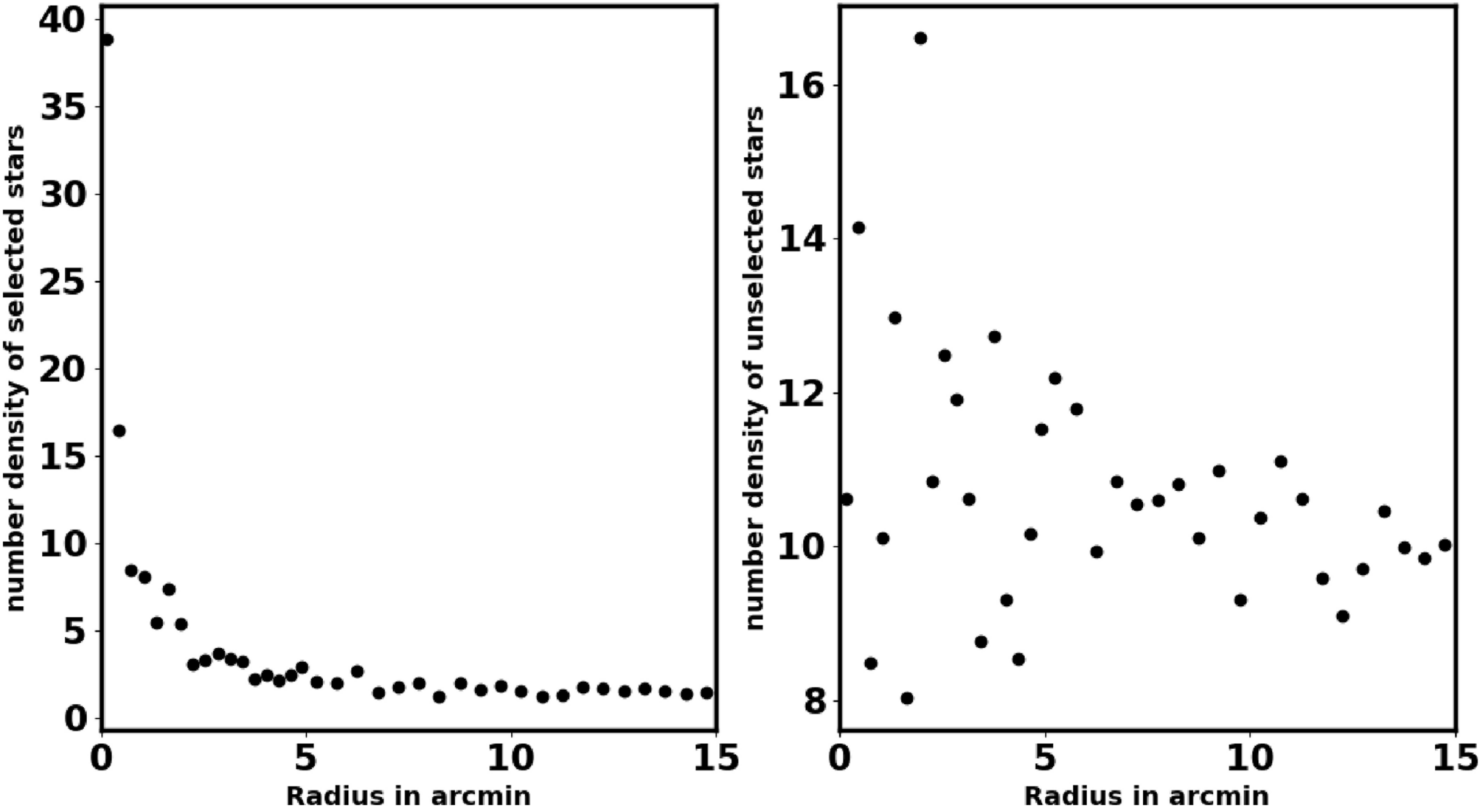
The left panel: the number stars density of selected stars, in case of the parallax condition $0.2 \le \varpi \le 0.4$, while the right panel is RDP of unselected stars.

When working with Gaia data, it is crucial to accurately cut off the data to avoid significant problems and incorrect conclusions about cluster properties, such as the count of member stars, core radius, mass and overall size. The King model helps us to distinguish between background noise and actual member stars, allowing for minimal data clipping. For our analysis, we restricted the Gaia data to a parallax range of 0.05 to 0.9 milliarcseconds (mas). The data clipping process can lead to a decrease in the number of member stars and field stars together. For example, in Fig. 3, we illustrate the radial density profile (RDP) of stars that do not meet the selection criteria, specifically those with parallax values between 0.2 and 0.4. This figure indicates that the clipped data is mostly an over-density of member stars. *It is important to analyze the RDP of these unselected stars to assess if any distinct structures or concentrations of stars still exist in the data.* But if we calculate the average proper motions and parallaxes, we use Gaussian fit to determine these averages, as shown in Fig. 8 in section "Proper motions, cluster kinematics and dynamics".

## Cluster density profile

The initial step in analyzing the cluster structure and creating the radial density profile is to precisely identify the center of the cluster. Our main goal is to identify the greatest concentrated area inside the cluster. We created a two-dimensional histogram displaying star counts in both directions, right ascension ($\alpha$) and declination ($\delta$), using the Gaia DR3 database. We used the ‘histogram2 d‘ function from the NumPy package (https://numpy.org/.) to determine which cell has the highest number of stars. This process was repeated in Section "Membership determination", focusing solely on member stars, and we found no significant differences.

To assess the extent of the cluster in the sky, we create the radial density profile (RDP) of NGC 2509 by segmenting the observed area into concentric rings. The number of stars ($N_i$) and the area of each ring ($A_i$) are computed, and the star density is calculated as $f_i = N_i / A_i$, where the i-th ring area $A_i = \pi (r_{i+1}^2 - r_i^2)$. The total density function $f_{t}(r)$ for the field and members is defined as:

1 $$\begin{aligned} f_{t}(r)= f_{bg} + f_{c}(r) \end{aligned}$$

where $f_{bg}$ and $f_{c}(r)$are the background density and the cluster’s member density, which is defined by^28^.

2 $$\begin{aligned} f_t(r) = \Bigg \{ \begin{array}{lcc} f_{bg} \;+\; k \times \left[ \dfrac{1}{\sqrt{1+(r/r_c)^2}} \;-\; \dfrac{1}{\sqrt{1+(r_t/r_c)^2}} \;\; \right] ^{\beta } & , & \text {if } r \le r_{t}\\ f_{bg} & , & \text {if } r> r_{t} \end{array} \end{aligned}$$

where $\beta$ is typically 2, but we set it to 1 to achieve lower errors and more reasonable radius values than using $\beta = 2$.

*k* is related to the central density $f_o$ as follow:

3 $$\begin{aligned} k \;=\; f_o \times \left[ 1 \;-\; \dfrac{1}{\sqrt{1+(r_t/r_c)^2}} \right] ^{-\beta } \end{aligned}$$

$r_c$, and $r_t$ are the core and tidal radii of the cluster, respectively. The core radius $r_c$ is the distance from the cluster center at which the stellar density is equal to :

4 $$\begin{aligned} f_c(r_c) \;=\; k \times \left[ \frac{1}{\sqrt{2}} \;-\; \frac{1}{\sqrt{1+(r_t/r_c)^2}} \right] ^{\beta } \end{aligned}$$

The tidal radius $r_t$ is the distance far from the cluster center at which cluster stars density is equal to zero. On this concept, $f_{c}(r_i)$ denotes the density of fitting member stars in the (${i}$) ring, then the number of members in that ring is given by $f_{c}(r_i) \times A_{i}$, where the radius of that ring calculated as:

5 $$\begin{aligned} r_i \;=\;(r_{i}+r_{i+1})/2 \end{aligned}$$

Another important parameter we have constructed is the number of member stars which can be specified as follows:

6 $$\begin{aligned} N_{cl}= \sum _{r=0}^{r=r_t} \left[ N_i - f_{bg}\; A_i \right] \end{aligned}$$

This value depends on $r_t$. *Moreover, it will be used to constrain the probability cut-off value as we will see in the next section.*

**Fig. 4:**
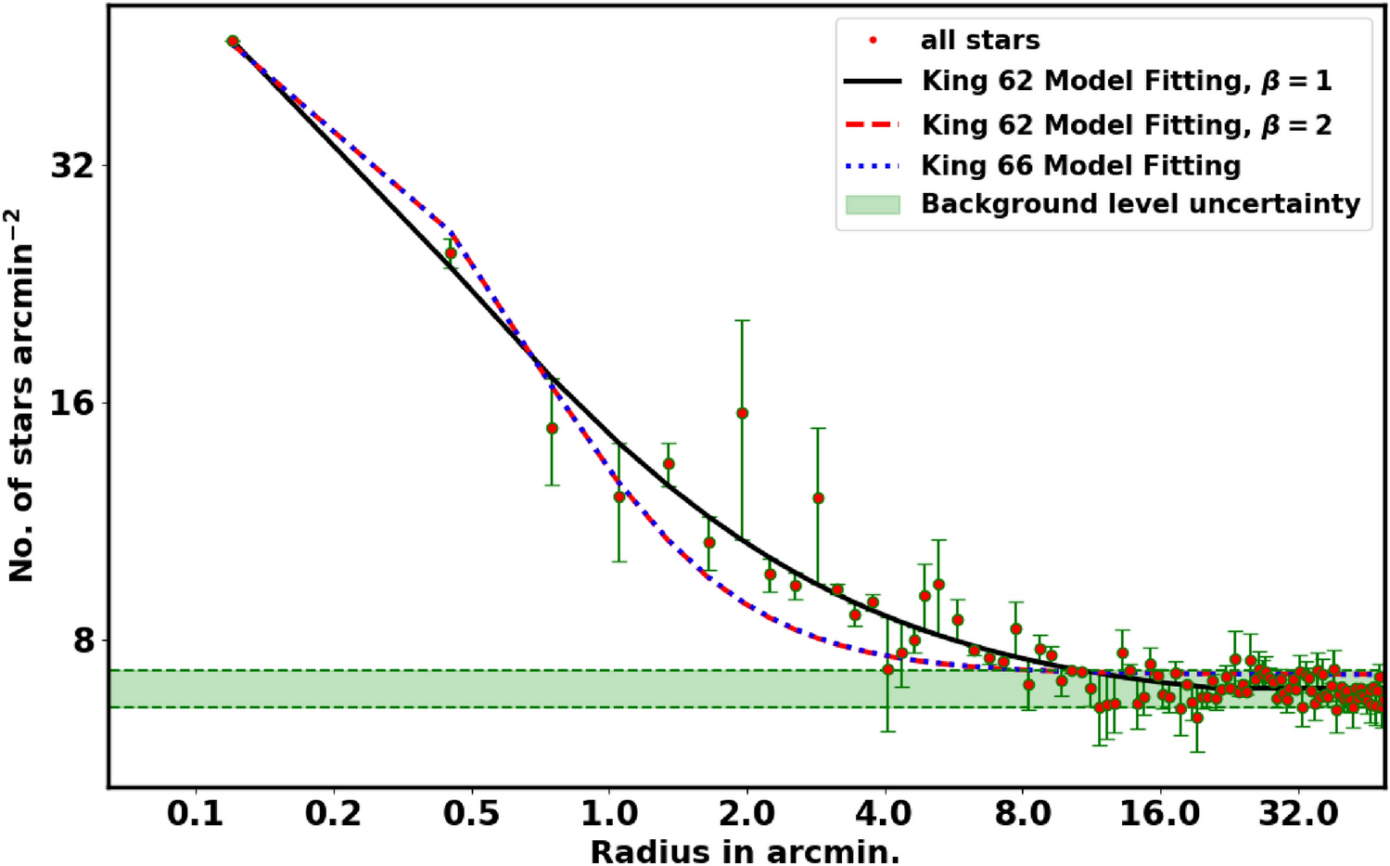
The radial density profile (RDP) of NGC 2509.

Fig. 4 represents the fitting of the King models to the RDP of NGC 2509. The best fit in equation 2 occurs when $\beta$ equals 1, but it is not a general case. The green dashed line indicates the background density, $f_{bg}$, which is found to be $9.23 \pm 0.18$stars $\text {arcmin}^{-2}$. The computed values for the central density $f_o$, core radius $r_c$, and tidal radius $r_{t}$ are $51.95\pm 5.25$stars $\text {arcmin}^{-2}$, $0.16 \pm 0.04$arcmin, and $19.25\pm 3.25$arcmin, respectively, see Table 2. Moreover, $N_{cl}$ is found as $497 \pm 39$stars. The uncertainties in the fitted parameters are estimated using the covariance matrix from the curve_fit function in the Scipy package (https://scipy.org/.).

In literature, there is anther method to determine the cluster radius. The limiting radius, $r_{lim}$, is determined by comparing the cluster’s density with the surrounding background density, i.e., the radius at which the cluster’s RDP becomes stable concerning the background density. It is derived by^29^ as:

7 $$\begin{aligned} r_{lim} = r_c \sqrt{\frac{f_o}{3 \sigma _{bg}} - 1} \end{aligned}$$

where $\sigma _{bg}$ is the uncertainty of $f_{bg}$. For NGC 2509, $r_{lim}$ is found to be approximately $11.5 \pm 0.87$arcmin which is smaller than $r_t$.

**Table 2:** The result of radial density profile (RDP) fit with equation2.

$f_o$	$f_{bg}$	$r_c$	$r_t$	Ref.
stars$\text {arcmin}^{-2}$	stars$\text {arcmin}^{-2}$	arcmin	arcmin	
$51.95\pm 5.25$	$9.23 \pm 0.18$	$0.16 \pm 0.04$	$19.25\pm 3.25$	this work
32.334±3.236	15.816±0.511	0.578±0.113	-	^25^

Finally in this section, the star density contrast, $\delta _c$, is a crucial parameter for the cluster, which is expressed as:

8 $$\begin{aligned} \delta _c = 1 + \frac{f_o}{f_{bg}} \end{aligned}$$

For NGC 2509, the contrast parameter is $6.63 \pm 0.02$, which is in the range of ($7 \le \delta _c \le 23$) as reported by^30^, indicating that NGC 2509 is a sparse cluster.

## Membership determination

The determination of fundamental parameters for star clusters is often complicated by contamination from field stars. Historically, cluster membership was determined using photometric and kinematic data^31–33^. However, with the advent of astrometric data from the Gaia survey, the accuracy of kinematic methods for membership determination has significantly improved. Proper motion and parallax data are particularly effective in distinguishing field stars from cluster members, as stars in a cluster tend to share similar kinematic properties and distances^34^.  In this work, we utilized Gaia DR3 proper motion and parallax data to differentiate cluster members from non-members.

**Fig. 5:**
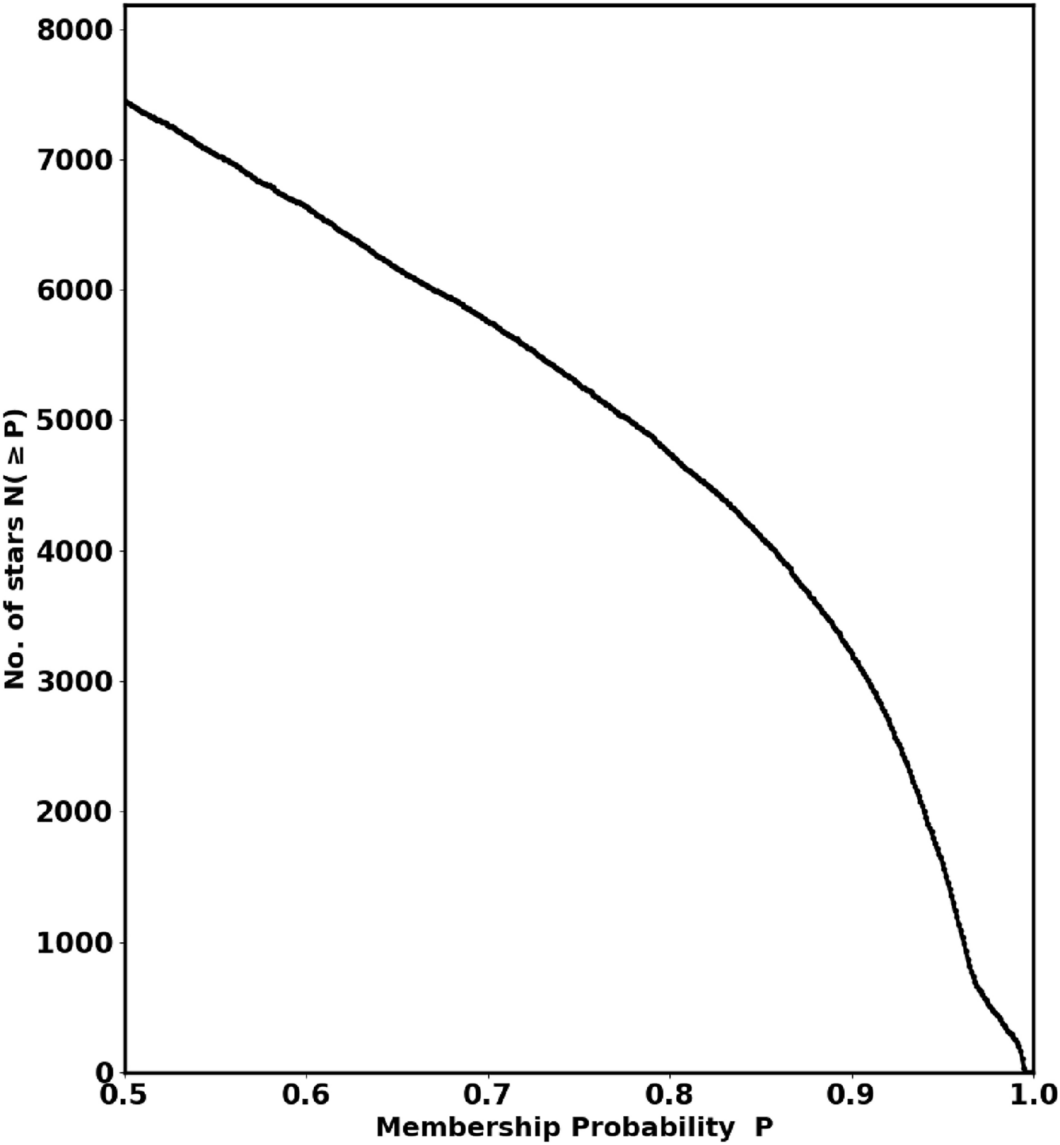
The number of stars $N(\ge P)$ as a function of membership probability, the output of *pyUPMask* code.

The Unsupervised Photometric Membership Assignment in Stellar Clusters (UPMASK) algorithm, developed by^35^, HDBSCAN method, is a non-parametric and unsupervised method that eliminates the need for prior field star selection. A refined version, available as the *pyUPMASK* Python package (https://github.com/msolpera/pyUPMASK.)^36^, extends the original algorithm by incorporating several clustering methods from the scikit-learn library^37^ (https://scikit-learn.org/stable/.), (https://github.com/adamdempsey90/StarClusters), enabling more flexible analysis of unlabeled data. In this study, we employed the *pyUPMASK* package to calculate membership probabilities for stars within the cluster. Gaia DR3 data for approximately 58,791 stars within a $40^\prime$ radius were used as input. Fig. 5 shows the total number of stars, N($\ge$P), as a function of their probability of membership (P). Based on a King profile fit (Section "Cluster density profile"), we identified $497 \pm 39$cluster members more accurately.

### The probability cut-off value

In many cases, a 50% probability cut-off is used to determine whether a star belongs to the cluster. However, this isn’t always the best approach. The optimal cut-off value depends on the method used and factors like the density of stars around the cluster and the star’s distance from the center. Furthermore, different clusters may require different probability cut-offs. It’s important to thoroughly test the chosen cut-off because using an incorrect threshold can wrongly classify stars as members or not. Additionally, the fitted King profile model significantly affects this classification process.

The optimal cut-off value varies based on the method used and factors like star density around the cluster and a star’s distance from the center. Different clusters may necessitate distinct probability cut-offs. It’s crucial to rigorously test the selected cut-off, as an incorrect threshold can lead to misclassifying stars as members or non-members. Moreover, the fitted King profile model significantly influences this classification process.

The threshold value for probabilities is a topic of ongoing discussion and varies across recent studies. For instance^38^, applies the HDBSCAN method with a probability cut-off of 50%. In contrast^39^, employs UPMASK with P $> 70\%$, while^40^ uses the GMM model with $\hbox {P}> 80\%$. Generally, there are two types of probability cut-off values: (i) an integrated value for the entire cluster and (ii) a value applied at each ring, or shell. Our new methodology sets a cut-off value at every shell of radius $r_i$, as detailed in equation 5 and equation 6.

9 $$\begin{aligned} Nm_{i}(P\ge P_i) \;\approx \; f_{c}(r_i) \; A_i \end{aligned}$$

where $P_i$ probability in *i-th* shell which gives the number of member stars as $Nm_{i}$ and must equal the number of stars from King model, $f_{c}(r_i) \; A_i$. We re-plotted the stellar density profile for the member stars only, as shown in Fig. 7. The results are highly satisfactory and align well with the King profile.

**Fig. 6:**
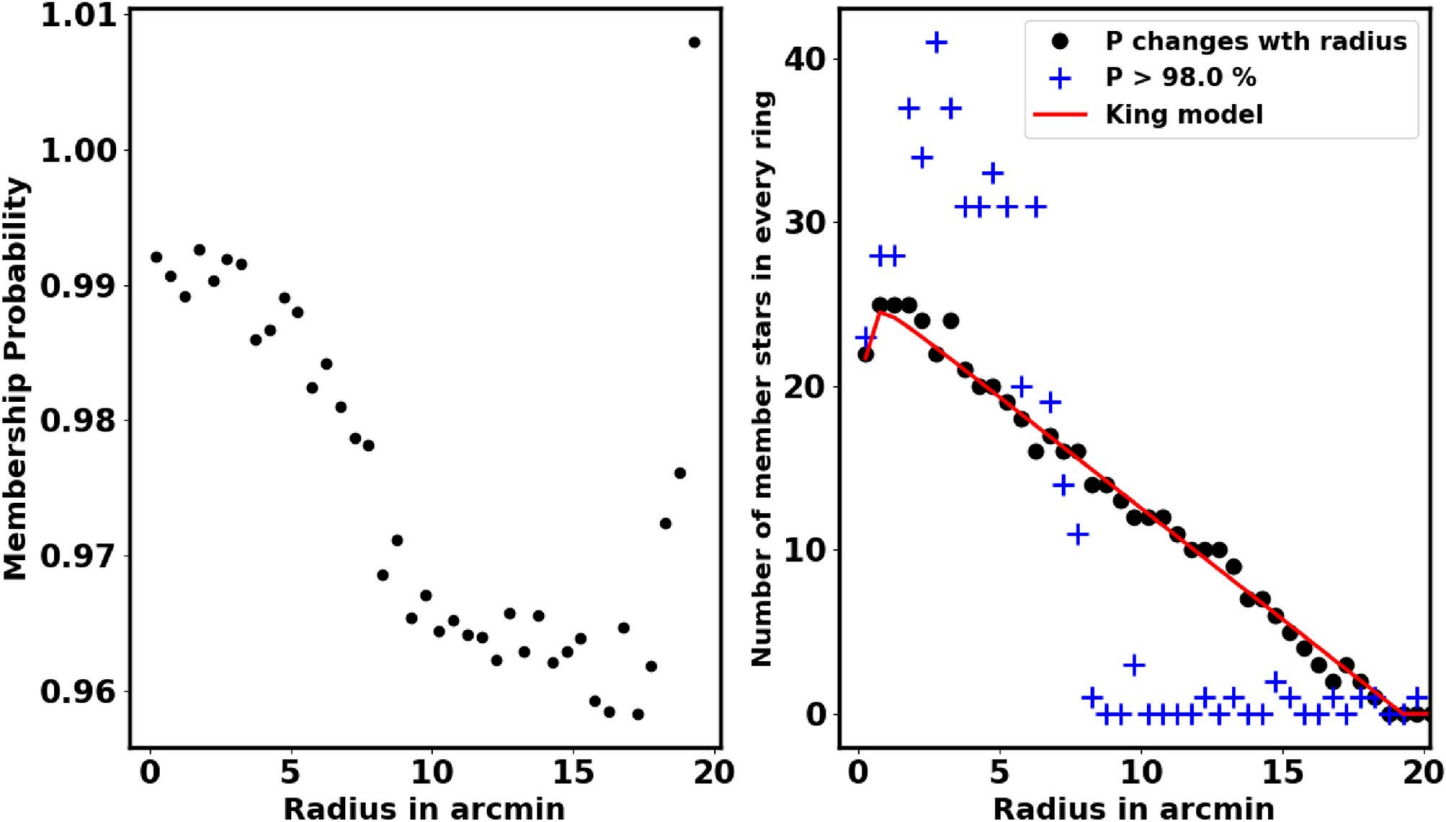
The graph illustrates how membership probability varies with the shell’s radius in arcmin. **The left-hand panel** focuses on determining membership probability based on each shell’s radius. **In the right-hand panel,** the blue crosses indicate members with a probability greater than 98% for the entire cluster, leading to overestimated member counts in some part and underestimated in other part. The black dots indicate the number of members with the radius based on our current approach of varying membership with radius, while the red line is number of members inferred from the Kind model fit.

Moreover, in Fig. 6, the right-hand panel shows the one value of membership probability for whole cluster, which exceeds 98%. *This singular probability value results in an overestimation in certain areas while leading to an underestimation in others.* We modified this approach to assess membership probability based on the radius of each shell, as illustrated in the right-hand panel of that figure. Our results align closely with the King fit model for each shell. The blue crosses indicate members with a probability greater than 98% for the entire cluster, resulting in overestimated member values. We can get the index of these members in python as :

10 $$\begin{aligned} ind_{memb,i} = \left( P>= P_i \right) \end{aligned}$$

The King density profile is a useful tool for verifying the accuracy of the membership separation method and the total number of cluster members. Conversely, an incorrect membership separation method or probability cut-off can cause overestimation or underestimation of member stars.

**Fig. 7:**
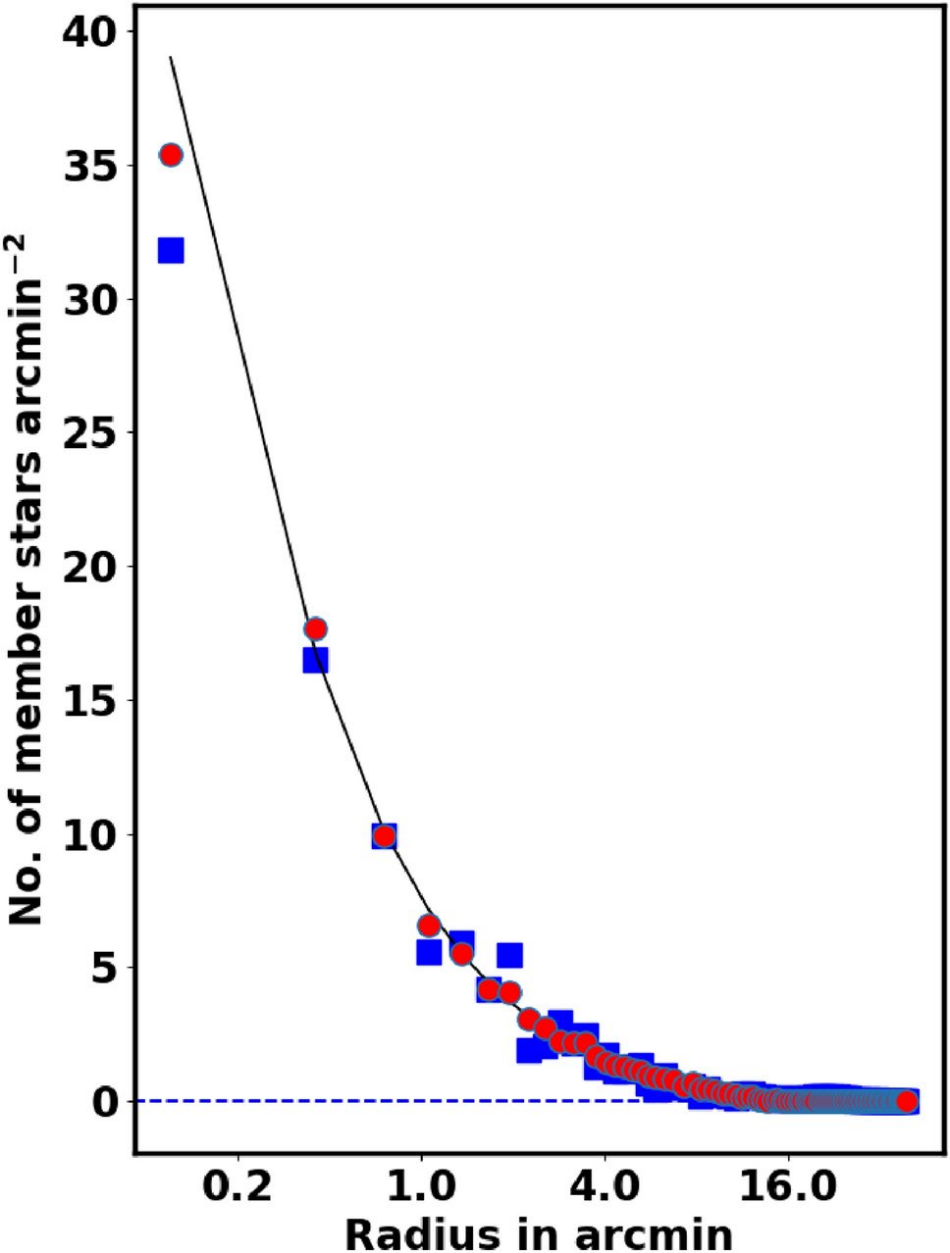
The stellar density profile of the member stars is depicted, with the solid line representing the fitted King profile from Equation 2. The red dots indicate the most probable member stars, while the blue squares correspond to member stars collected from^19^.

## Proper motions, cluster kinematics and dynamics

For more than 1.3 billion sources, the third Gaia data release (Gaia DR3) offers precise five-parameter astrometric data (positions, proper motions, and parallaxes). These vast amounts of data help us to understand the kinematic and evolutionary evolution of open clusters with unprecedented precision and accuracy, thanks to Gaia DR3. The center of the cluster is located at $120.21 \pm 0.06^\circ (8:0:49.0)$and $-19.06 \pm 0.06^\circ (-19:03:15.1)$, which corresponds to the Galactic coordinates l=$237.84 \pm 0.06^\circ$and b=$5.84 \pm 0.06^\circ$.

**Fig. 8:**
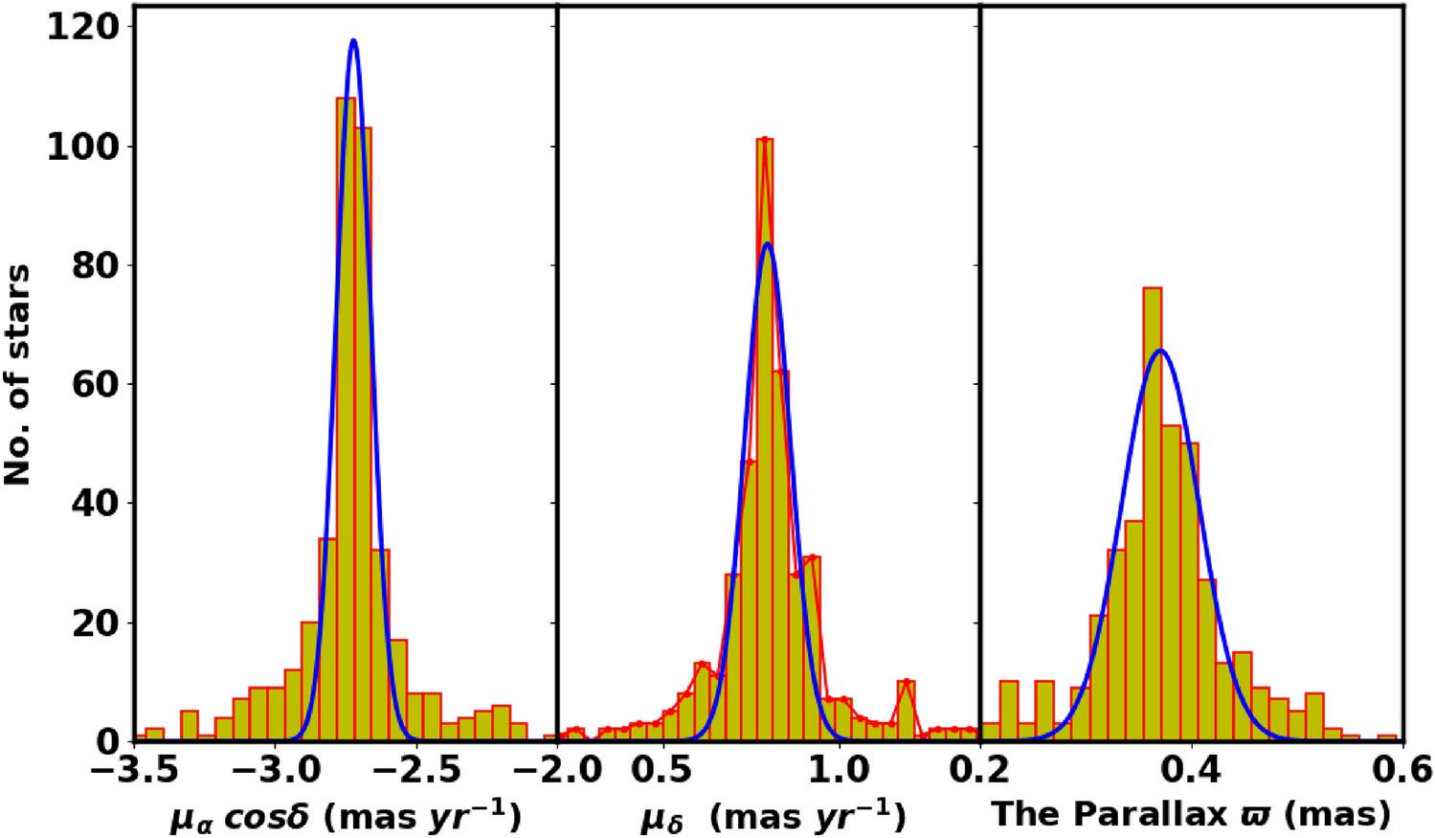
The proper motions and parallaxes histograms of the member stars are illustrated. The blue lines are representing the Gaussian fits.

To accurately determine the cluster parameters, we fit the proper motions and parallaxes of members with Gaussian distribution. The mean value of proper motions components are $\mu _{\alpha } \cos \delta =$ $-2.710 \pm 0.142$ $\text {mas yr}^{-1}$ and $\mu _{\delta } =$ $0.802 \pm 0.146$ $\hbox {mas yr}^{-1}$, see Fig. 8. The mean value of parallax ($\varpi$) is found to be $0.368 \pm 0.043$mas. The corresponding distance to the cluster, calculated as $d_{\varpi } (\text {pc}) \approx 1000 / \varpi (\text {mas})$, is $2638.008 \pm 230.5$kpc. This value is consistent with the results obtained from photometric data within the associated uncertainties, see Fig. 8. The detailed results are presented in Table 1.

**Fig. 9:**
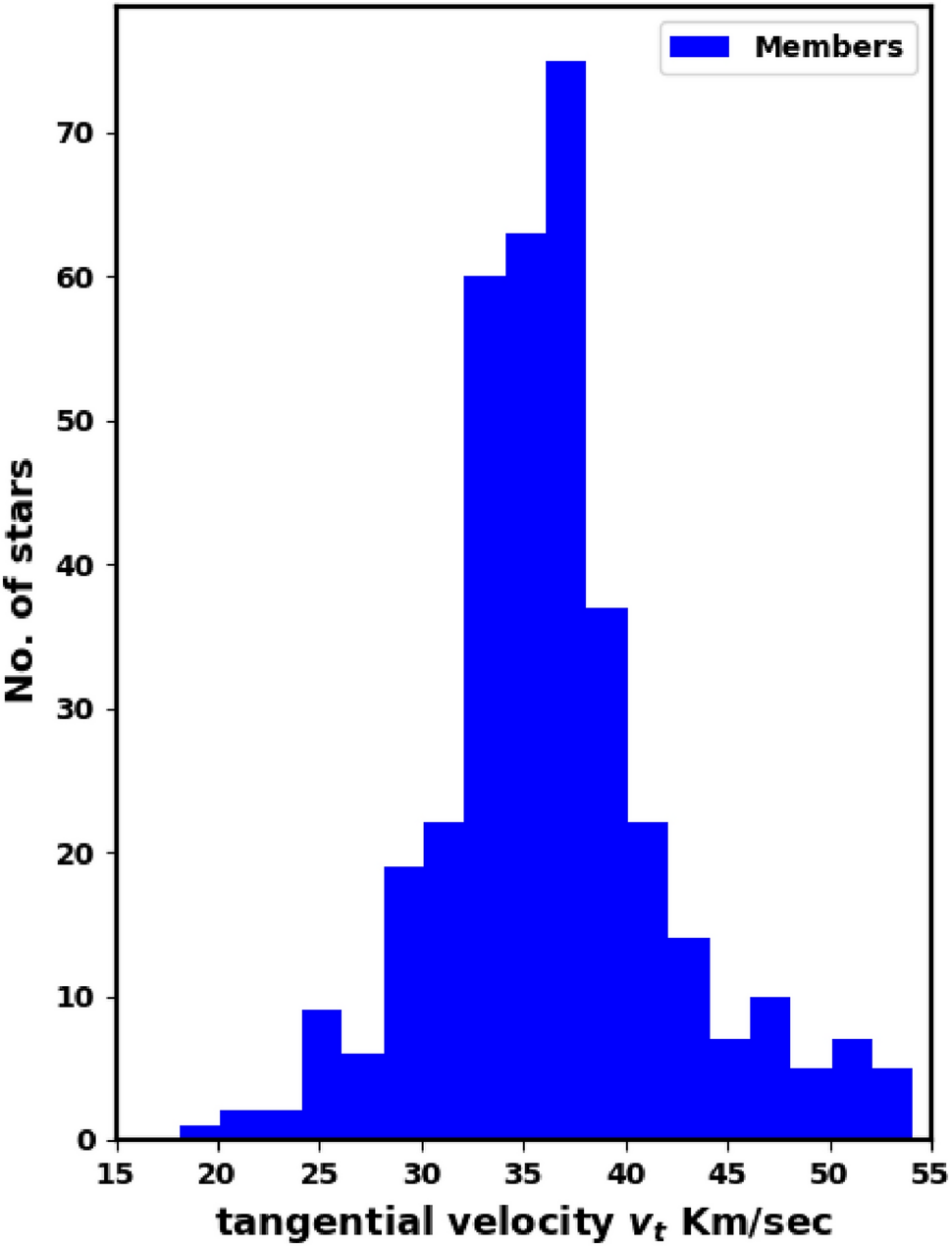
Tangential velocities histogram of member stars with average $35.7 \pm 6.2$km/s.

Tangential velocities of open clusters derived from absolute proper motions and parallaxes tied directly to galaxies allow one to determine the orbit type of the studied cluster, which contributes to a substantial improvement in studies of cluster origin and destruction processes. Tangential velocity in km/s unit is:

11 $$\begin{aligned} v_t = 4.47 \;\dfrac{\mu }{\varpi } \end{aligned}$$

where $\mu$ and $\varpi$ are proper motion and parallax in unit of mas $yr^{-1}$ and mas. The Fig. 9 shows tangential velocity $v_t$ histogram with an average value of as $35.7 \pm 6.2$km/s, nearly Gaussian distribution.

Determining the tangential velocity alone is not sufficient; we need to know its direction. So, another key parameter is the angle $\theta$, which indicates the direction of the cluster’s motion in the $\mu _{\alpha } \cos \delta$ and $\mu _{\delta }$ space (Fig. 11), which is described as:

12 $$\begin{aligned} \theta = \tan ^{-1}\left( \frac{\mu _{\delta }}{\mu _{\alpha } \cos \delta } \right) \end{aligned}$$

Cluster member stars generally move in nearly the same direction through space; see^41^. Fig. 12 presents a histogram of $\theta$ for member stars, with an average angle of $162.49 \pm 7.95^\circ$, providing a clearer view compared to Fig. 11. Additionally, the dispersion in the $\theta$ histogram may reflect the cluster’s age and its degree of gravitational binding.

**Fig. 10:**
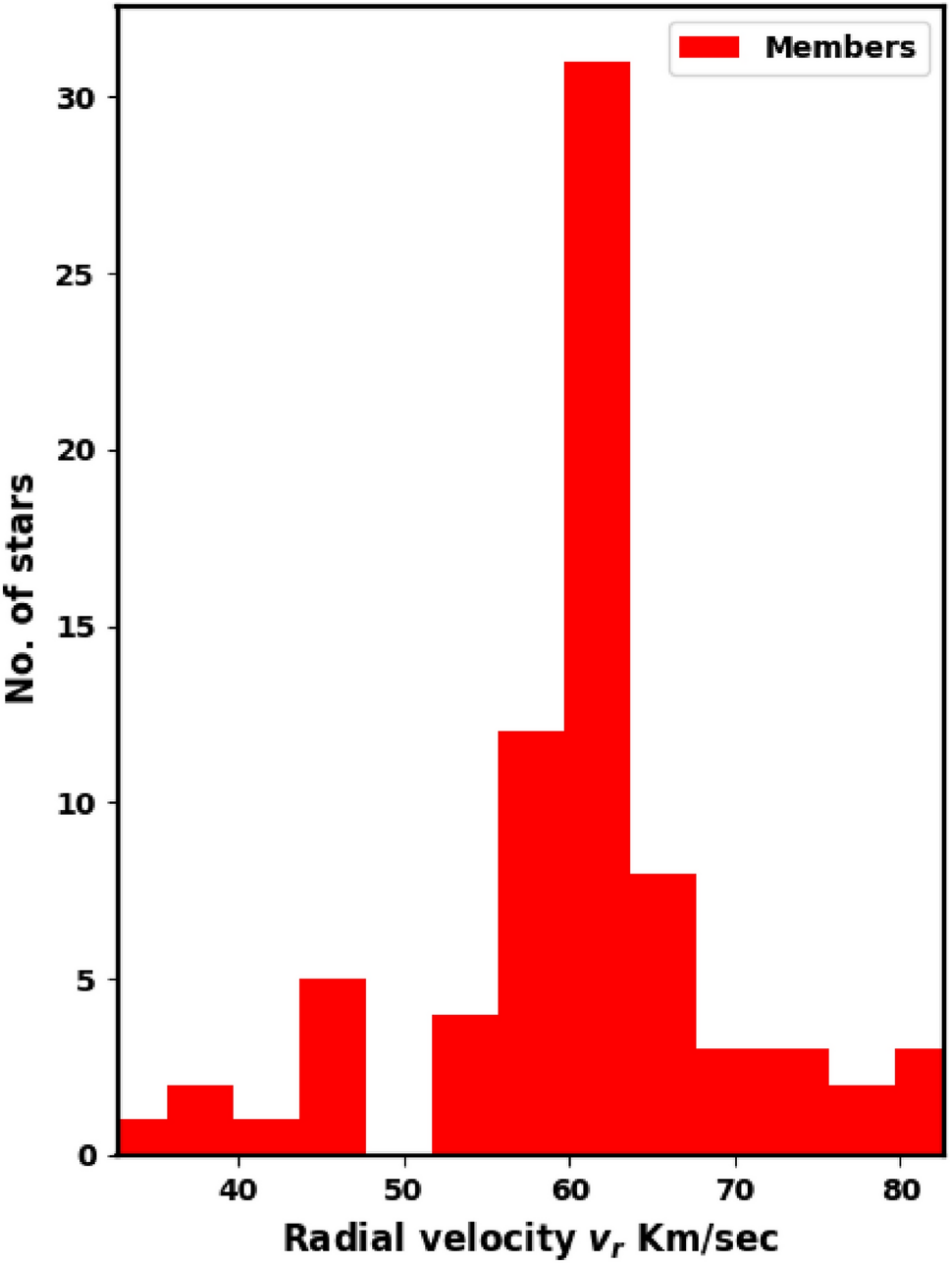
Radial velocities histogram of member stars with average $57.6 \pm 7.8$km/s.

Moreover, there are 88 member stars that have radial velocities in Gaia DR3 with an average value of about $57.6 \pm 7.8$km/s, see Fig. 10. This value is in agreement with the value 58.71 km/s of^19^and the value 60.83 km/s of^42^. Therefore, the space velocity of the cluster ($v_{space} = \sqrt{v_r^2 + v_t^2}$) is approximately $68.9 \pm 11.1$km/s, making an angle of about $56.2 \pm 5.6^{\circ }$ with a tangential velocity direction. Then we can get the cluster orbit parameter; see the next section.

**Fig. 11:**
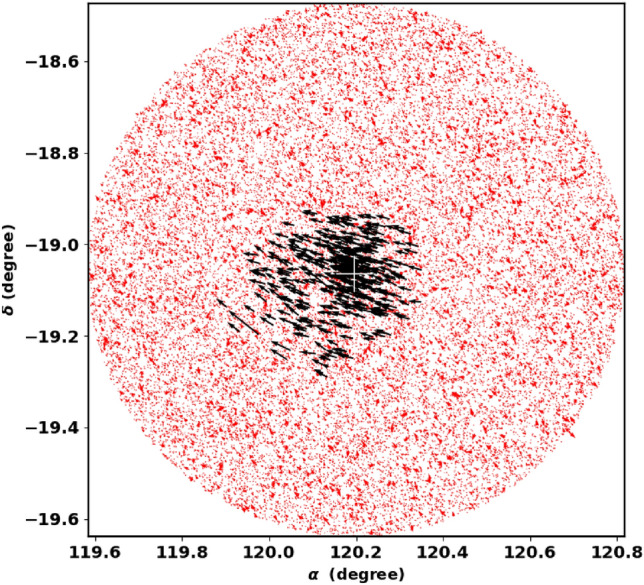
The co-moving stars of NGC 2509, which indicates that the members have almost the same direction in the sky.

**Fig. 12:**
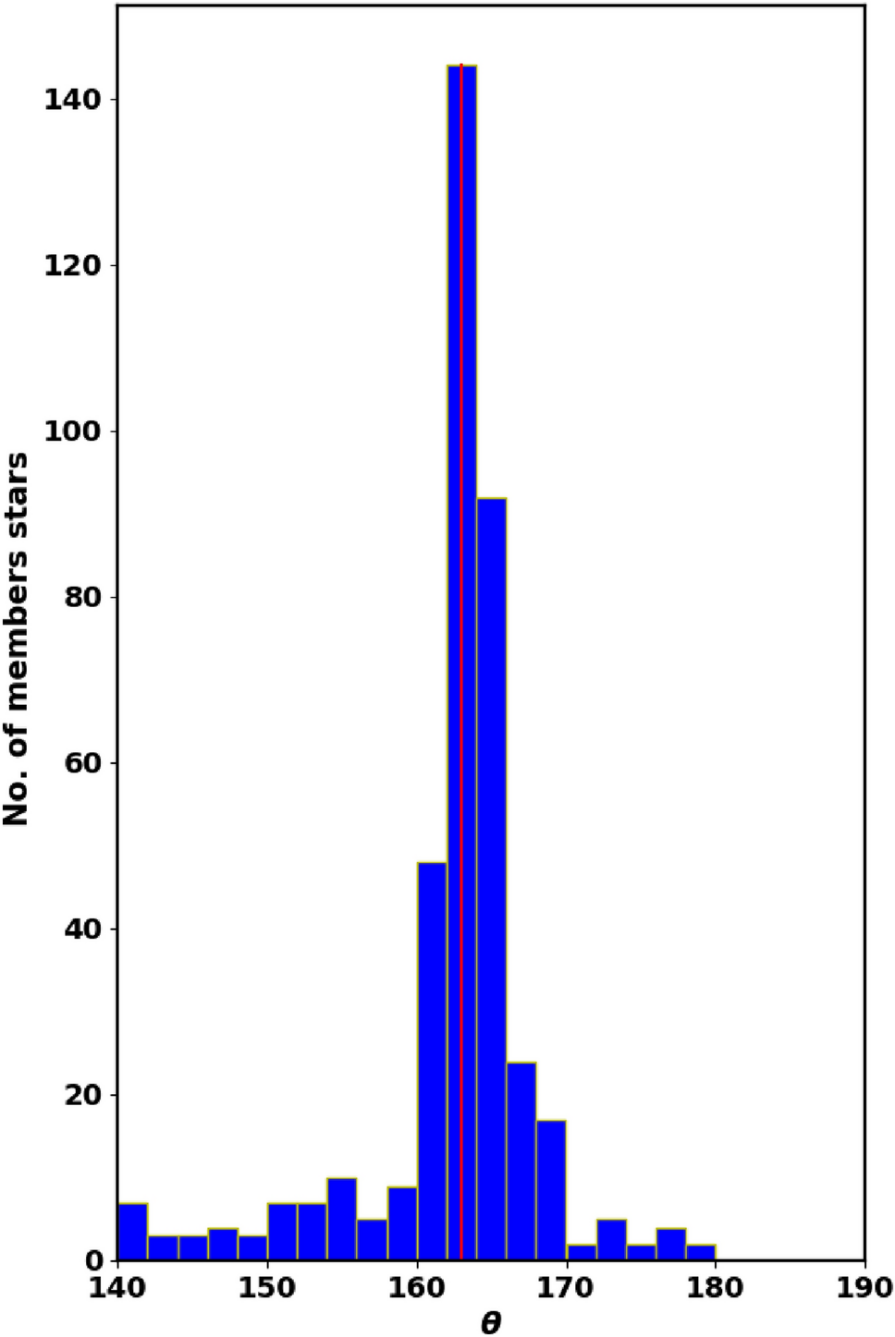
The $\theta$ histogram for member stars, which indicates the direction of the cluster’s motion in the $\mu _{\alpha } \cos \delta$ and $\mu _{\delta }$ space.

### Cluster orbit

OCs are very good tracers of the evolution of the Galactic disc. Thanks to **Gaia DR3**, their kinematics can be investigated with unprecedented precision and precision of proper motion and parallax ($\mu _{\alpha } \cos \delta$, $\;\mu _{\delta }$ and $\varpi$). Moreover, Gaia DR3 provides the radial velocities (RV) of many million relatively bright, late-type stars^43^collected by the Radial Velocity Spectrometer (RVS) instrument^44^. The combination of parallax, proper motion, and RV gives access to phase-space information. As examined^45^, illustrated the great potential of Gaia data for studying the kinematics of the Galactic disc, where OCs revealed the richness of phase-space substructures.

OCs ages reflect the full lifespan of the Galactic disc, from young to old thin-disc components. Their spatial distribution and motion enhance our understanding of the Galaxy’s gravitational potential and the perturbations affecting its structure and dynamics. The orbital motions of OCs are crucial for understanding their dynamical evolution in the Galaxy and for studying Galactic dynamics.

To compute any cluster orbit, it is necessary to adopt first a model for Galaxy potential. This potential must produce the observed mass density of the Galaxy. So, we have performed backward orbital integration of NGC 2509 using **“MWPotential2014”**potential the default galpy (^46^) potential of the Milky Way. This potential model, a three-component model, includes bulge, disk, and halo potentials. **(1)**The Galactic disk potential is defined by Miyamoto-Nagai expression (^47^), **(2)**the bulge component is described as spherical power-low potential (^46^) and **(3)**finally, the Navarro-Frenk-White dark matter halo potential^48^. The Sun’s galactocentric, orbital velocity and z were taken as $R_{GC}=8$ kpc, $V_{rot}=220$ km $s^{-1}$, and z =20.8 pc. As an input, we have used the cluster parameters, i.e., presented PMs ($\mu _{\alpha }cos\delta$, $\mu _{\delta }$), distance from the Sun, equatorial coordinate ($\alpha$, $\delta$) and radial velocity, which was calculated as an average from the Gaia DR3 data of member stars.

**Fig. 13:**
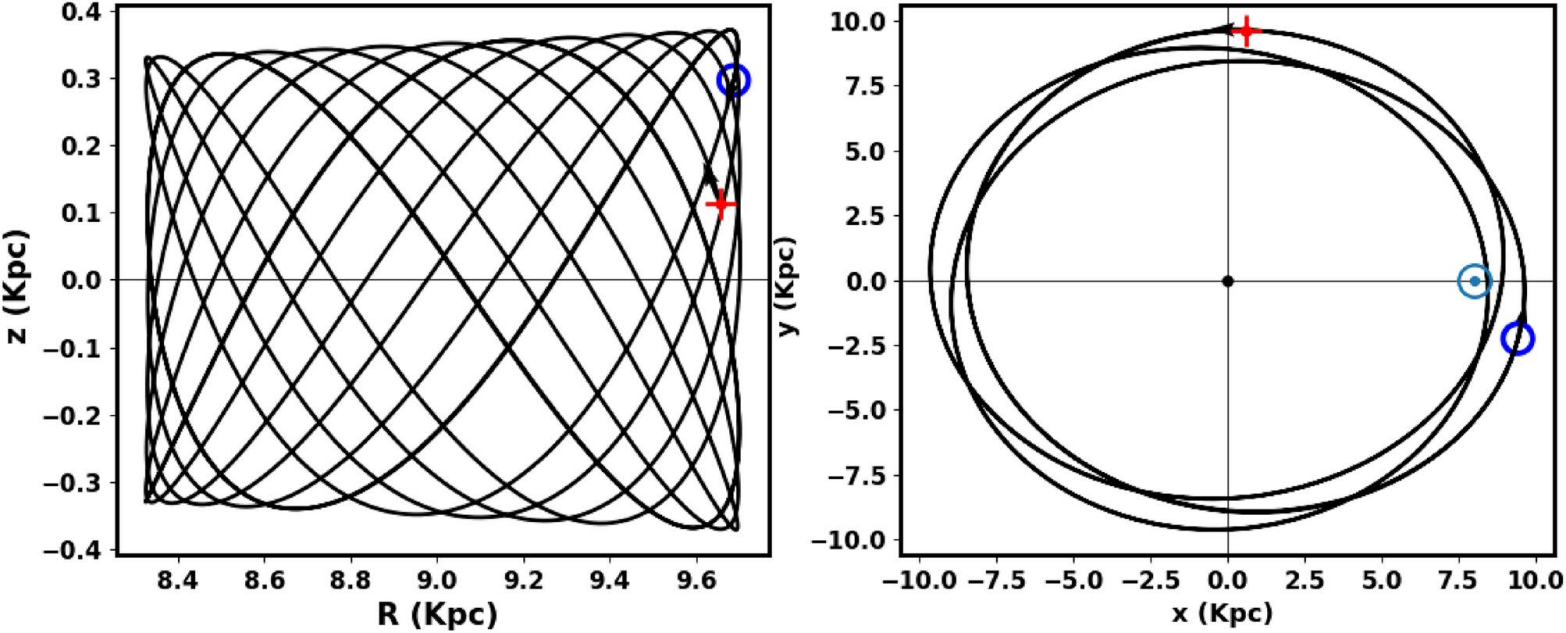
The cluster orbit. The red cross is the birthplace.

**Fig. 14:**
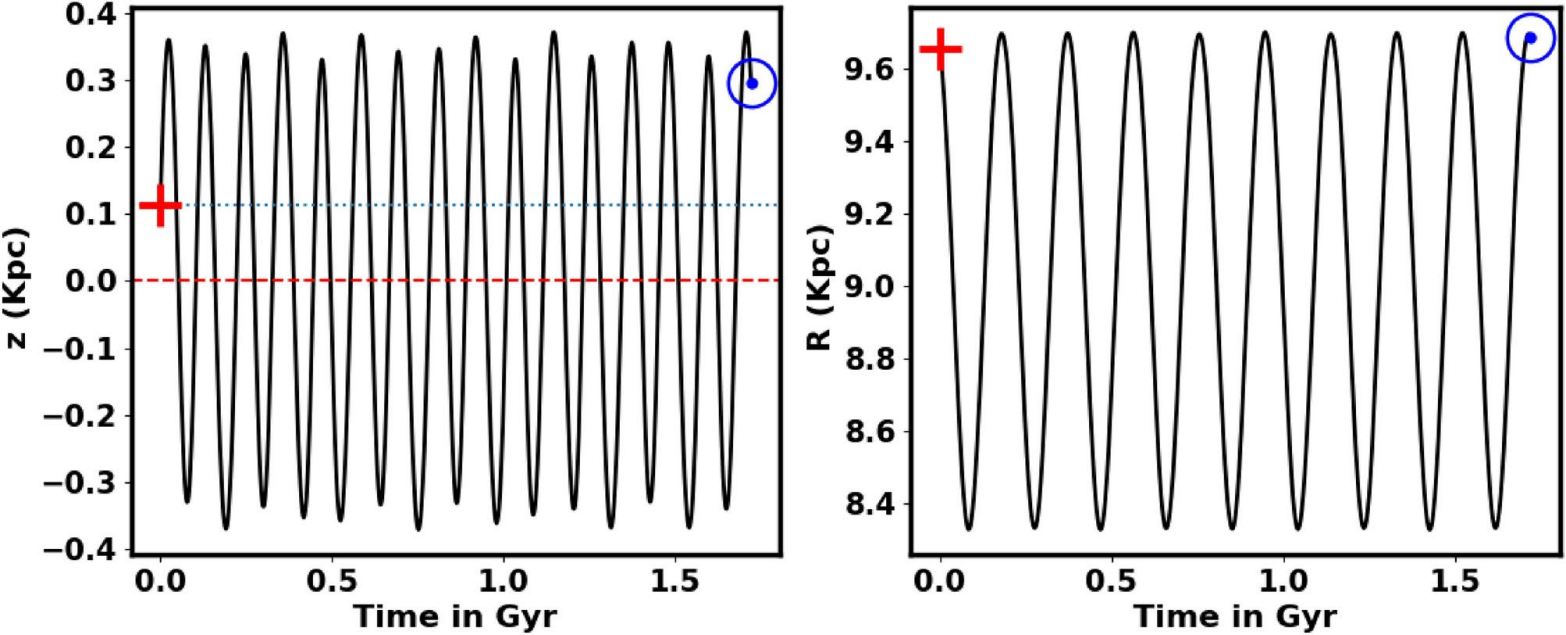
The NGC 2509 cluster completes 15.5 revolutions around the Galactic plane $1.72\pm 12.3$.

**Fig. 15:**
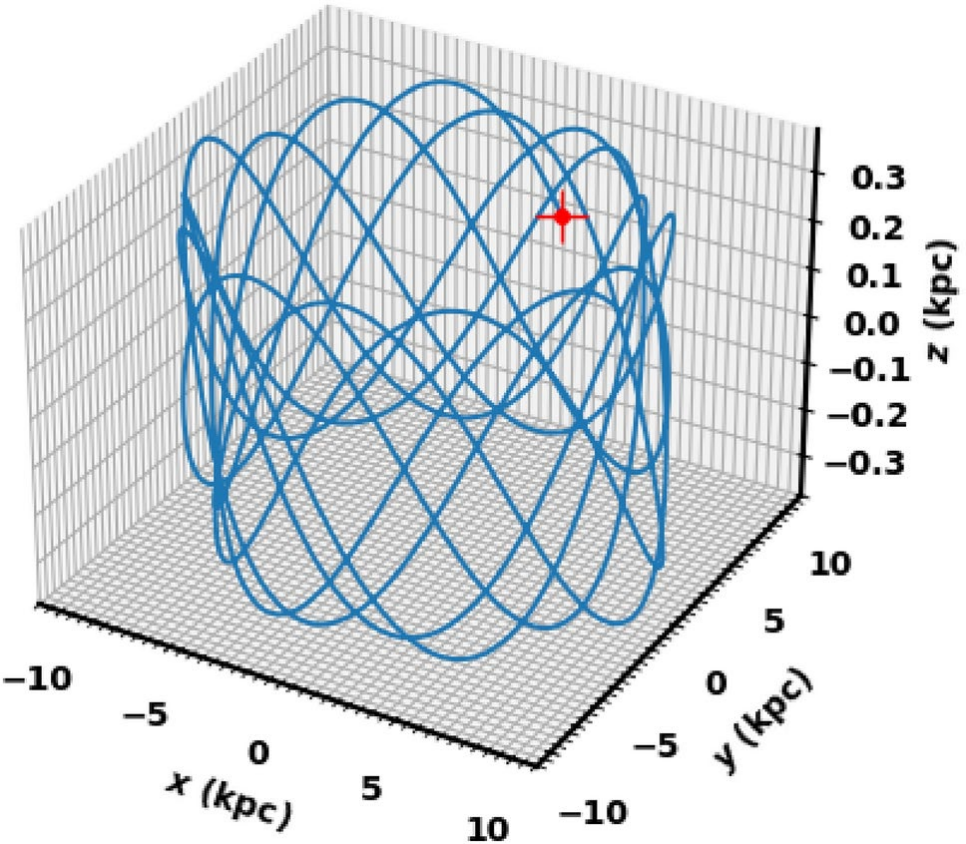
The cluster’s orbit in 3D Cartesian Galactocentric coordinates, with the red cross marking its birthplace.

Figs. 13 and 15 show the integrated orbit of NGC 2509 in the Cartesian Galactocentric coordinate system backward in time according to the age of the cluster determined in this paper. The red cross is the birthplace of the cluster. According to the z coordinate, the cluster oscillates approximately more 15 times around the Galactic plane, rising to a maximum height of 363.17 pc (see Fig. 14). Thus, NGC 2509 is part of the Galaxy’s thin-disk component. Its orbital eccentricity is about e= 0.069 ±0.012, with vertical and radial periods (Tz and Tr) of 0.11 Gyr and 0.19 Gyr, respectively. Refer to Table 3 for the results of the orbital parameters and their comparison with other data.

**Table 3:** The orbital’s parameters.

Radial vel.	Apocenter	Pericenter	Eccentricity	$R_{gal}$	$T_r$	vx	vy	vz	U	V	W	Ref.
km/s	kpc	kpc		kpc	Gyr	km/s	km/s	km/s	km/s	km/s	km/s	
$57.6 \pm 7.8$	9.70	8.44	0.069	9.69	0.19	44.90	196.76	−10.17	$-$54.5	$-$36.4	$-$18.2	this work
60.83	9.82	8.13	0.094	9.81	–	$-$42.17	211.59	$-$8.0	–	–	–	^42^
60.83	–	–	–	–	–	–	–	$-$9.58	$-$54.55	$-$39.97	$-$16.83	^49^
58.71	–	–	–	–	–	–	–	–	–	–	–	^19^

### Mass distribution inside cluster radius

To study cluster dynamics, we need to know the mass distribution with the cluster radial profile. Over time, two-body relaxation causes more massive stars to migrate toward the cluster’s center, while less massive stars tend to occupy a larger volume and gradually evaporate. If this were true, we would anticipate finding a concentration of massive stars in the center of this intermediate-age cluster, but that is not the case. Furthermore, the mass distribution concerning the radial profile in NGC 2509 provides a different viewpoint.

**Fig. 16:**
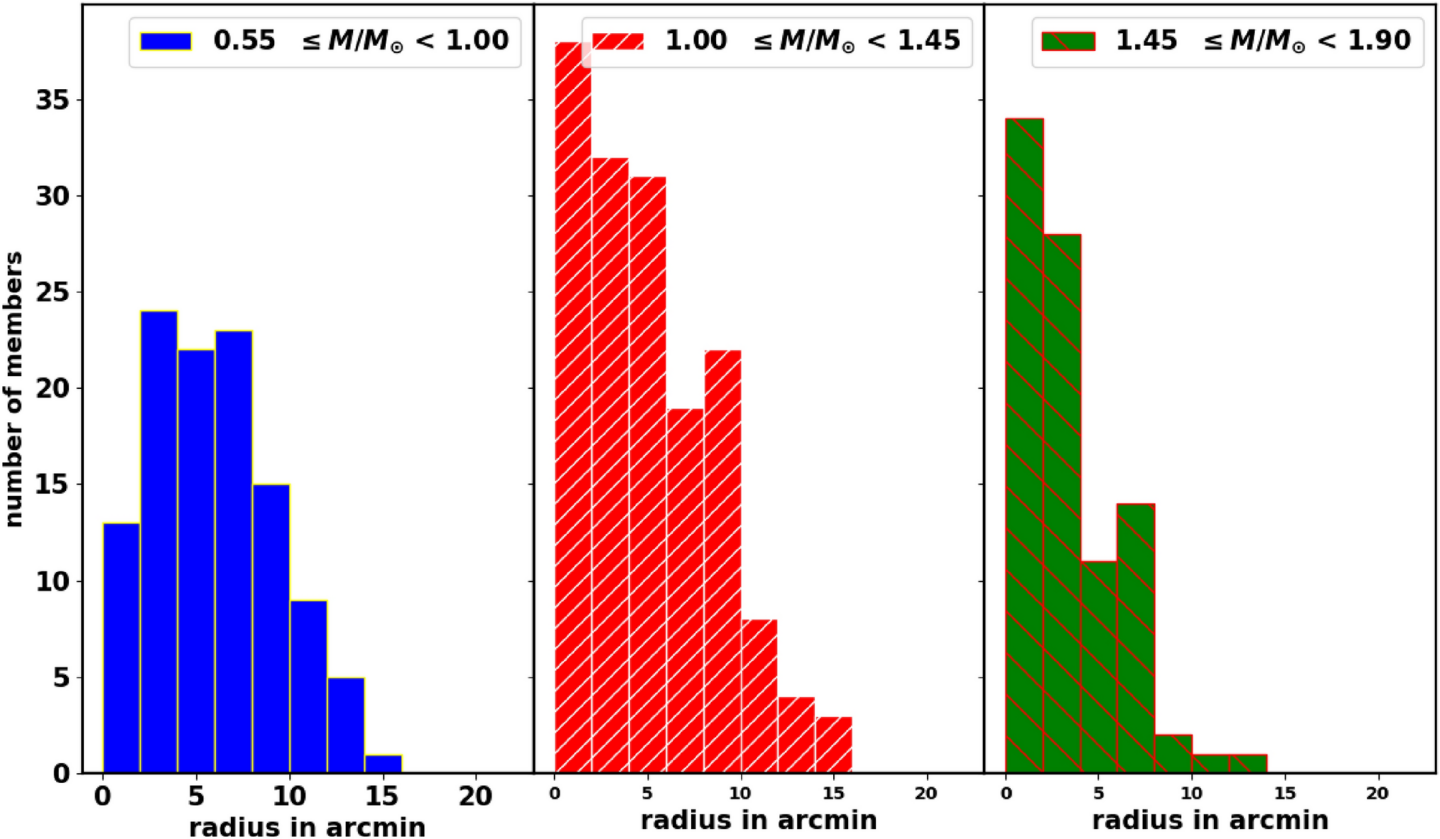
The mass distribution of the cluster’s members across radial bins.

We divide the members into three mass intervals and count the stars in each radial bin, as shown in Fig. 16. Our findings indicate that the mass distribution concerning the radius is similar across all three intervals. This means that the drift from the cluster is influenced by stellar position rather than mass. Therefore, the stars at the outskirts of the cluster tend to drift away and eventually dissolve, while those in the core remain more tightly bound by cluster gravity and are less affected by Galactic tides. Thus, the displacement of member stars depends on gravitational acceleration rather than their masses.

### Dynamical state

A key factor in understanding the dynamical evolution of a star cluster is the relaxation time. This is the time required for the cluster to lose its initial conditions and for the member stars to approach a Maxwellian velocity distribution. According to^50^, the relaxation time can be described as:

13 $$\begin{aligned} T_R = \dfrac{8.9 \times 10^5 \sqrt{N} \times R_{h}^{1.5}}{\sqrt{m} \times \log (0.4N)} \end{aligned}$$

where *N* is the number of cluster members, $R_h$ is the radius (in parsecs) containing half of the cluster’s total mass, and *m* represents the average stellar mass (in solar units).

In Fig. 21, we plot the mass $M(>r)$ inside radius r. From this figure, we find that the value of $R_h$ is equal to $4.3 \pm 0.85$arcmin. Then, By applying the above equation, we found that the relaxation time of the cluster is equals $93.5 \pm 24.5$Myr which is much younger than the cluster age $1.72\pm 12.3$. That means NGC 2509 is dynamically stable and a relaxed cluster.

## Photometric analysis of the cluster

### Color magnitude diagram

Generally, Color-magnitude diagrams (CMDs) for open clusters utilize empirical isochrones for comparison with theoretical models of stellar evolution^51,52^. CMD is a worthy tool for estimating the important key parameters, e.g., age, distance, reddening (color excess), and metallicity of the studied cluster. Moreover, by comparing the observed CMD with theoretical isochrone, significant information regarding the mass of the members in the cluster can be acquired. The theoretical isochrones utilized in this study were downloaded from the CMD 3.7 website (http://stev.oapd.inaf.it/cgi-bin/cmd.), using PARSEC version of 1.25 s^53^.

The interstellar dust extinction law is crucial for interpreting observations accurately. Extinction coefficients for different pass-bands are influenced by the spectral energy distribution of the source, interstellar matter, and the extinction itself. The relative extinction $A_\lambda /A_{\lambda _1}$ and the color excess ratio E($\lambda -\lambda _1$)/E($A_{\lambda _2}-A_{\lambda _1}$) both serve as indicators of the extinction law^54^.

We compute the extinction coefficient in the Gaia pass-bands comparing to the optical bands as follows: $A_G/A_V=0.789$, $A_{BP}/A_V= 1.002$, $A_{RP}/A_V=0.589$, where $\text {A}_{V} = 3.1 \times E(B-V)$. We can then establish the connection between extinction and color excess in the present form: $\text {A}_{G} = 1.84 \times E(G_{BP} - G_{RP})$. Isochrone fitting allows us to estimate the color excess and, subsequently, the extinction. Then, the intrinsic distance modulus $(m-M)_o$ can be calculated using the following equation:

14 $$\begin{aligned} \left( m-M \right) _{obs} = \left( m-M \right) _{o} + A_{G} \end{aligned}$$

**Fig. 17:**
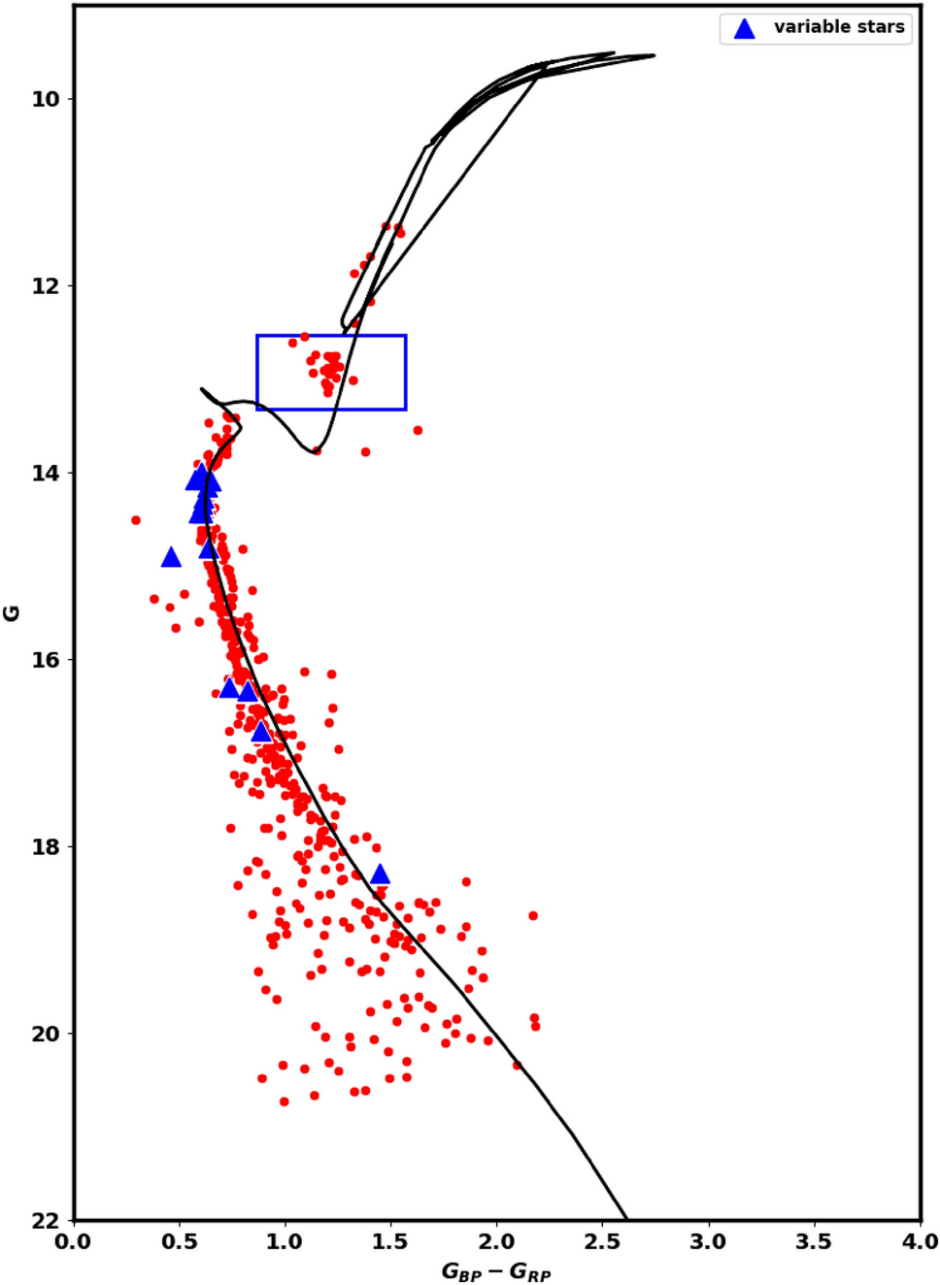
The color-magnitude diagram (CMD) for the member stars in the NGC 2509 cluster. It is created using data from the Gaia DR3 photometric bands: G, $G_{BP}$, and $G_{RP}$. In this diagram, blue triangles indicate stars identified as variable in Gaia DR3. The stars located within the rectangle on the diagram are classified as red clump stars (RC). See also Figure 18.

From the photometric data of Gaia DR3, the CMD of NGC 2509 is presented in Fig.17, applying the theoretical isochrones of^51^. The intrinsic distance modulus and the color excess are found to be $11.68\pm 0.12$mag and $0.13 \pm 0.04$mag, respectively. These results correspond to an isochrone-based distance $d_{iso}$ of $2168.5\pm 261.5$pc, which has been found to match closely with the value inferred from the parallax. The fitted isochrone indicates a cluster age of $1.72\pm 12.3$Gyr, with a metallicity of $\hbox {Z} = 0.0152$.

**Fig. 18:**
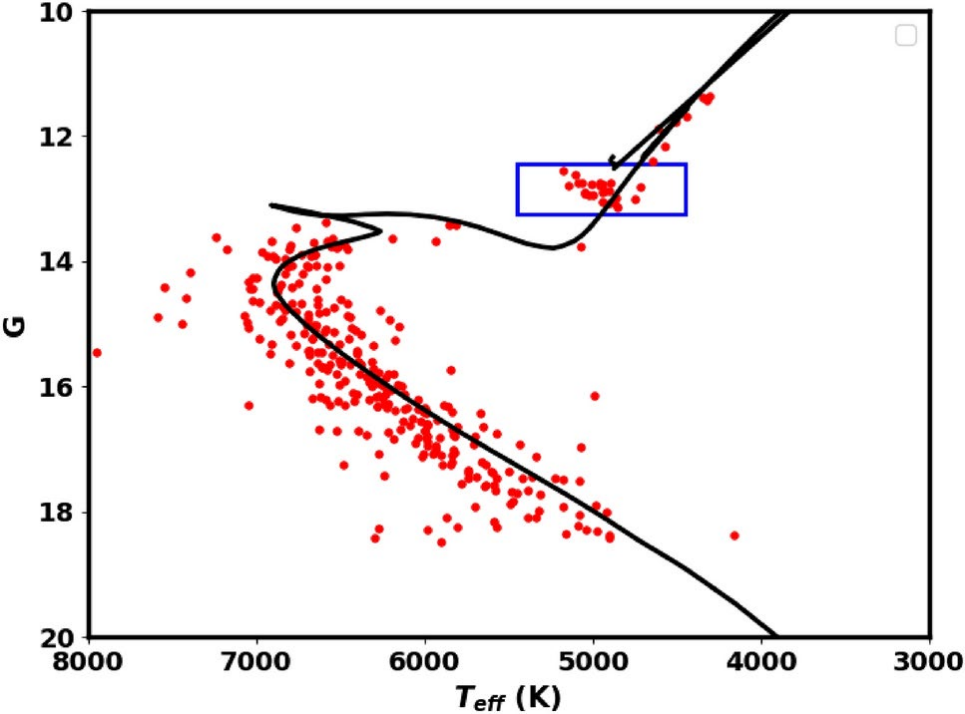
The plot displays the effective temperature ($T_{eff}$) on the horizontal axis versus the *G*magnitude on the vertical axis. The effective temperatures used in the plot are sourced from the work of^55^. The solid black line represents the best fit isochrone, which is consistent with the one of the same age and metallicity as shown in Fig. 17. The stars enclosed within the rectangle are identified as red clump stars (RC), similar to those marked in Fig. 17.

We obtained essential astronomical data, such as the effective temperatures from^55^, and created a plot showing the Gaia G magnitudes versus these temperatures, as shown in Fig. 18. The solid black line represents the same isochrone featured as used in Fig. 17. This plot constrains the metallicity and chemical composition of the cluster. Additionally, it is crucial to examine the evolved stars by understanding their positions within this diagram.

Red clump **(RC)**stars are commonly observed; evolved stars. They evolved and transitioned from sun-like stars to red giants, supported by helium fusion in their cores. They generally have similar absolute luminosity regardless of their age or composition, causing them to clump in a specific area of a color-magnitude diagram. This characteristic makes them useful as standard candles for astronomical measurements. The study by^56^ provides a comprehensive catalog of 2.6 million red clump stars. In the current study, we identified 20 member stars matched with this catalog, as illustrated in Fig. 17. These stars have G magnitudes between 12.6 and 13.1 mag and slightly higher temperatures than typical giants, as shown in Fig. 18.

### Luminosity, mass functions, and total mass

**Fig. 19:**
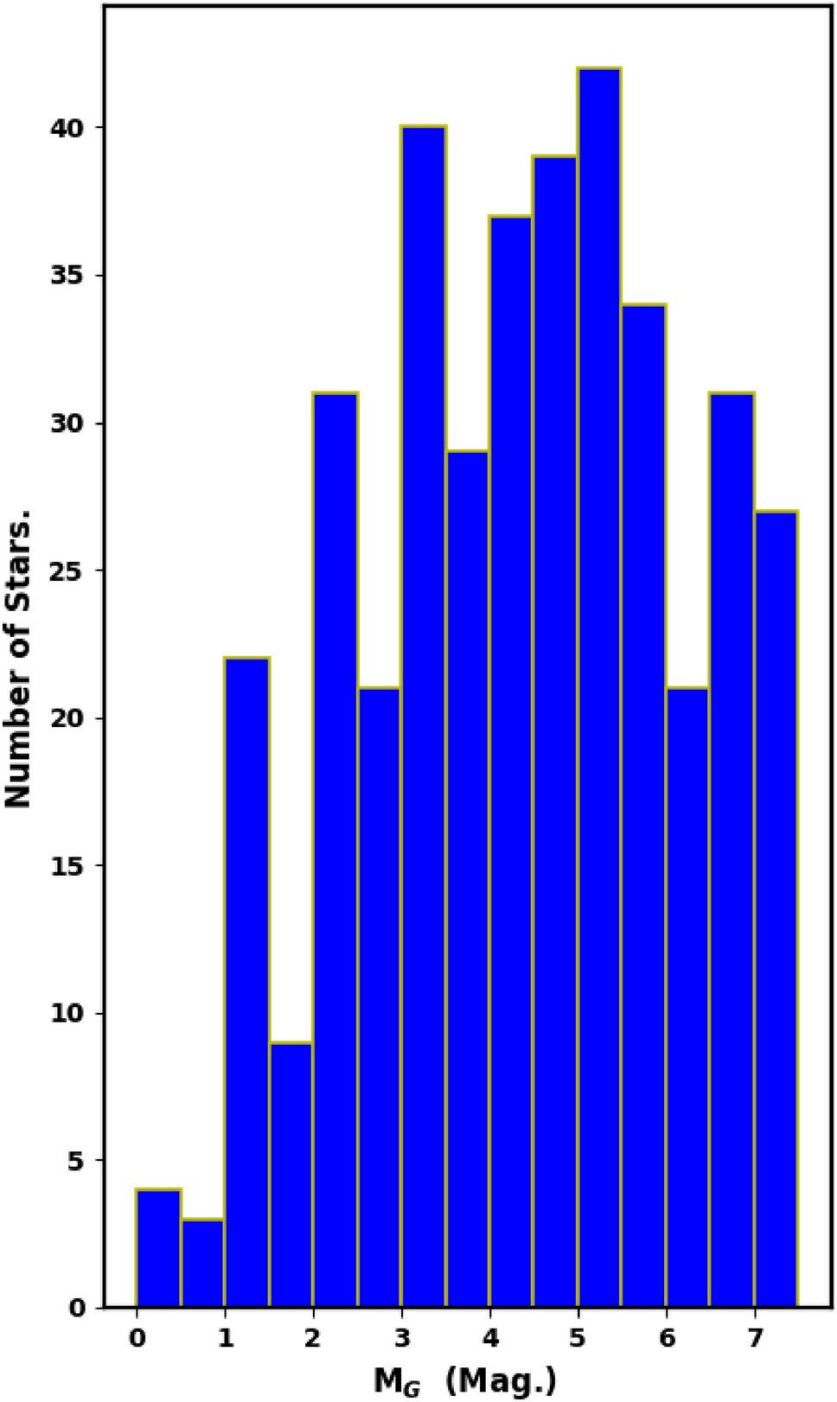
The luminosity function (LF) of NGC 2509, with a bin interval of 0.5 mag.

The luminosity and mass functions (LF and MF) are closely tied to the cluster’s membership. We employed the *pyUPMASK* Python package to identify probable cluster members in order to effectively remove contamination from field stars in the main sequence of NGC 2509. The apparent G-magnitudes of the member stars were converted into absolute magnitudes, and histograms were created to display the LF of NGC 2509 (Fig. 19).

The individual stellar masses of the cluster members are crucial for understanding the properties of the cluster. Following the isochrone fitting, we determined the absolute magnitude, $\text {M}_G$, and the intrinsic color, $G_{BP}-G_{RP}$for each member. The luminosity function (LF) can be converted into a mass function (MF) by using a mass-luminosity relation, usually obtained from theoretical models instead of direct observational transformations. To convert the LF to an MF, we used the theoretical isochrones from^51^and^57^. Thus, we employed an interpolation routine with two independent variables (the *SmoothBivariateSpline* function from the Python Scipy (https://scipy.org/.) package^58^), which allows interpolation with two variables, where a stellar mass is dependent on $M_G$ and $(G_{BP}-G_{RP})_o$. This approach enabled us to accurately determine the mass of each cluster member, yielding a total cluster mass of $\hbox {M}_c$ = $491.2\pm 59.5$ $M_\odot$. Additionally, we computed the cluster’s mass profile, as shown in Fig. 21, and derived the half-mass radius, $R_h$= $4.3 \pm 0.85$pc, where half of the total mass is required (see equation 13).

**Fig. 20:**
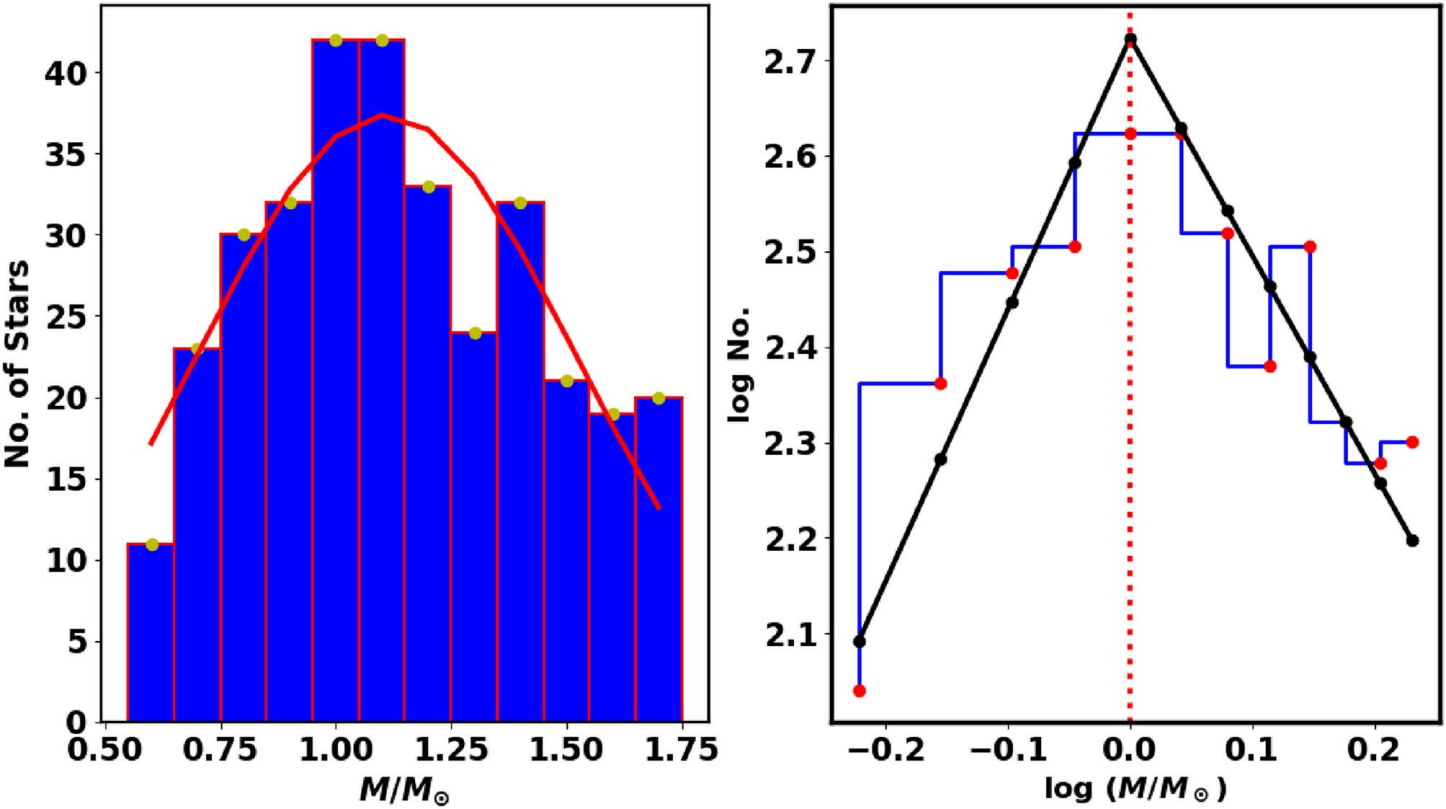
**The left panel:** displays the mass histogram with a Gaussian fit represented by a red line, featuring a mean of approximately 1.23 $M_{\odot }$ and a standard deviation $\sigma _{M}$ is 0.22. **The right panel:** presents the mass function (MF) of NGC 2509, where solid black lines indicate the low and high mass slopes of the mass function fits, with exponents $\alpha _1$ =$-2.74 \pm 0.14$, $\alpha _2=$ $2.29 \pm 0.13$and $M_{cr}=$ $1.1 \pm 0.17$, as described in equation 15.

In this study, we mathematically represent the mass function (MF) using a piecewise powerlaw that incorporates two distinct parts of the power law, as demonstrated by the work of^24^. This approach stands in contrast to the single power law equation that was originally proposed by^59^. Using this dual representation, we can gain a much deeper understanding of the mass distribution of stars during the initial phases of cluster formation, offering enhanced accuracy in our conclusions. This highlights the difficulty of stellar dynamics and the necessity for more precise models to accurately represent the diverse behaviors of stars in clusters as they evolve. The differences between the single and dual representations of the mass function reveal significant insights into how stars behave in various stages of their formation, providing essential information for future research in astrophysics. It can be represented as follows:

15 $$\begin{aligned} f(M) = \frac{dN}{dM} = \Bigg \{ \begin{array}{lcc} K_1 \; M^{-\alpha _1} & , & \text {if } M \le M_{cr}\\ K_2 \; M^{-\alpha _2} & , & \text {if } M> M_{cr} \end{array} \end{aligned}$$

under condition the function *f*(*M*) is continuous :

$$\begin{aligned} K_1 \; M_{cr}^{-\alpha _1} = K_2\; M_{cr}^{-\alpha _2} \end{aligned}$$

where dN/dM indicates the number of stars within the mass range *M* to $M + dM$. The parameters $\alpha _1$ and $\alpha _2$ represent the low and high mass slopes of the mass function, respectively, while $M_{cr}$ marks the critical mass where the slope changes in value and sign. The fitting is performed using the curve_fit function of the Scipy python package with $\alpha _1$, $\alpha _2$, $M_{cr}$, $K_1$, and $K_2$ treated as free parameters, constrained by the condition $f(M^{-}_{cr}) = f(M^{+}_{cr})$, within the mass range of 0.55 to 1.9 $M_{\odot }$. For NGC 2509, we found $\alpha _1$, $\alpha _2$, $M_{cr}$, $K_1$, and $K_2$ to be $-2.74 \pm 0.14$, $2.29 \pm 0.13$, $1.1 \pm 0.17$ $\; M_{\odot }$, $2.73 \pm 0.12$, and $3.0 \pm 0.17$(see Fig. 20). *Notably, the high mass slope* $\alpha _2$ *is close to the Salpeter value of 2.35*(^59^), *and the value of* $M_{cr}$ *corresponds to* $G \approx 16.56$ *Mag.*

**Fig. 21:**
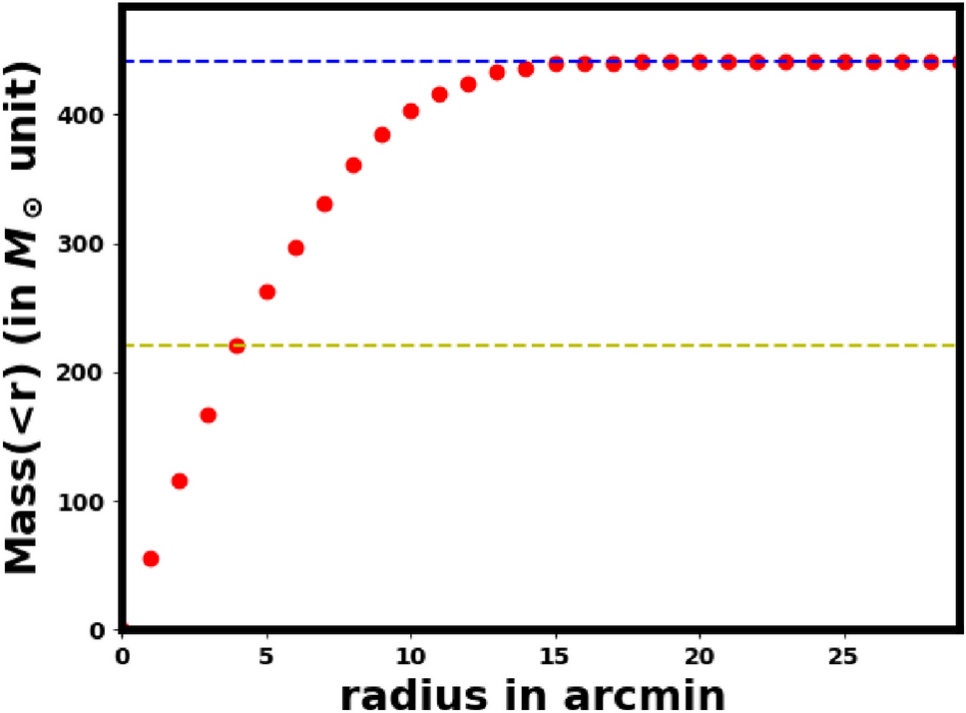
The mass profile $M(<r)$ of NGC 2509.   The horizontal blue dashed line indicates the total mass, while the yellow dashed line represents the half-mass radius, $R_h$.

## Summary and conclusions

We studied the middle-aged open cluster NGC 2509 using Gaia DR3 photometric and astrometric data to refine its fundamental parameters. Our main findings are as follows:

1) In NGC 2509, we identify $497 \pm 39$member stars with a total mass of $491.2\pm 59.5$. Based on Gaia DR3, we estimate the cluster’s age as $1.72\pm 12.3$Gyr, and its relaxation time as $93.5 \pm 24.5$Myr, indicating that NGC 2509 is a dynamically stable and relaxed cluster.

2) The distance modulus of the cluster derived from Gaia photometric CMD is $11.68\pm 0.12$mag, which corresponds to a distance of $2168.5\pm 261.5$pc. The color excess E($\text {G}_{BP}$-$\text {G}_{RP}$) is $0.13 \pm 0.04$mag^39^.

3) The proper motion values are ($\mu _{\alpha }\text {cos}\delta$, $\mu _{\delta }$), and parallaxes ($\varpi$) are $-2.710 \pm 0.142$mas, $0.802 \pm 0.146$mas and $0.368 \pm 0.043$mas/yr respectively. The cluster distance derived from the parallax ($\varpi$) is $2638.008 \pm 230.5$pc, which is consistent with the Gaia photometric CMD result within the errors. The cluster’s tangential velocity $v_t$ is $35.7 \pm 6.2$km/sec in the direction of $\theta =$ $162.49 \pm 7.95^\circ$, where $\theta$ is the angle between $v_t$ and $\mu _{\alpha }\text {cos}\delta$.

4) In the study of the mass function, we applied a piecewise powerlaw characterized by two power laws, which include five parameters: $\alpha _1$, $\alpha _2$, the critical mass $M_{cr}$, $K_1$, and $K_2$. The values we determined for these parameters are $-2.74 \pm 0.14$, $2.29 \pm 0.13$, $1.1 \pm 0.17$ $M_{\odot }$, $2.73 \pm 0.12$, and $3.0 \pm 0.17$, respectively.

4) Finally, we found that 11 members are classified as variable stars, and identified 20 member stars as red clump that have higher temperatures than the typical giants. In addition, there are 88 member stars that have radial velocities, which aid us to obtain the orbital parameters of NGC 2509.

## Data Availability

Gaia DR3 are available for free in webpage https://vizier.cds.unistra.fr/
